# Efficacy and acceptability of anti-inflammatory agents in major depressive disorder: a systematic review and meta-analysis

**DOI:** 10.3389/fpsyt.2024.1407529

**Published:** 2024-05-28

**Authors:** Yue Du, Yikai Dou, Min Wang, Yu Wang, Yushun Yan, Huanhuan Fan, Ningdan Fan, Xiao Yang, Xiaohong Ma

**Affiliations:** Mental health center and laboratory of psychiatry, West China Hospital of Sichuan University, Chengdu, China

**Keywords:** major depressive disorders, anti-inflammatory agents, anti-depressant activity, efficacy, acceptability

## Abstract

**Background:**

Anti-inflammatory agents have emerged as a potential new therapy for major depressive disorder (MDD). In this meta-analysis, our aim was to evaluate the antidepressant effect of anti-inflammatory agents and compare their efficacy.

**Methods:**

We conducted a comprehensive search across multiple databases, including PubMed, Embase, Web of Science, Cochrane Review, Cochrane Trial, and ClinicalTrials.gov, to identify eligible randomized clinical trials. The primary outcome measures of our meta-analysis were efficacy and acceptability, while the secondary outcome measures focused on remission rate and dropout rate due to adverse events. We used odds ratio (OR) and 95% confidence interval (95% CI) to present our results.

**Results:**

A total of 48 studies were included in our analysis. In terms of efficacy, anti-inflammatory agents demonstrated a significant antidepressant effect compared to placebo (OR = 2.04, 95% CI: 1.41–2.97, p = 0.0002). Subgroup analyses revealed that anti-inflammatory agents also exhibited significant antidepressant effects in the adjunctive therapy subgroup (OR = 2.17, 95% CI: 1.39–3.37, p = 0.0006) and in MDD patients without treatment-resistant depression subgroup (OR = 2.33, 95% CI: 1.53–3.54, p < 0.0001). Based on the surface under the cumulative ranking curve (SUCRA) value of network meta-analysis, nonsteroidal anti-inflammatory drugs (NSAIDs) (SUCRA value = 81.6) demonstrated the highest acceptability among the included anti-inflammatory agents.

**Conclusion:**

In summary, our meta-analysis demonstrates that anti-inflammatory agents have significant antidepressant effects and are well-accepted. Furthermore, adjunctive therapy with anti-inflammatory agents proved effective in treating MDD. Among the evaluated anti-inflammatory agents, NSAIDs exhibited the highest acceptability, although its efficacy is comparable to placebo.

**Systematic Review Registration:**

https://www.crd.york.ac.uk/prospero/display_record.php?RecordID=422004), identifier CRD42023422004.

## Introduction

Major depressive disorder (MDD) is a severe mental illness with a high incidence, disability rate, and suicide rate, causing significant harm to individuals, families, and society (1). During the COVID-19 pandemic, a review published in Lancet reported a 28% increase in the incidence of MDD, and the prevalence of major depressive disorder has been found to be associated with rising cases of SARS-CoV-2 infection (2). This suggests that inflammation could emerge as a prominent factor contributing to the development of MDD in the near future. Treating MDD effectively has encountered numerous challenges, and one of the most notable is that approximately one-third of individuals diagnosed with MDD fail to respond satisfactorily to multiple antidepressant medications (3). Furthermore, patients undergoing treatment often encounter side effects such as gastrointestinal symptoms and reduced libido, impacting treatment adherence and increasing the risk of suicide (4). Consequently, there is an urgent need for new treatments that can enhance effectiveness and safety.

In recent years, an increasing number of researchers have recognized the neuroinflammation hypothesis of MDD. Mase et al. (5) proposed that MDD is a neuropsychiatric disorder characterized by neuroimmune dysregulation, where inflammatory factors released due to peripheral immune activation directly influence depression-related neuroendocrine and behavioral changes. MDD patients have shown elevated levels of inflammatory cytokines, such as interleukin-6 (IL-6) and tumor necrosis factor-alpha (TNF-α), and the severity of depressive symptoms has been observed to correlate with the altered levels of inflammatory cytokines (6, 7). Previous researches have indicated that altered levels of pro-inflammatory cytokines lead to the activation of indoleamine 2,3-dioxygenase (IDO), an enzyme that metabolizes tryptophan into kynurenine, reducing serotonin production. Additionally, activated microglia promote the conversion of kynurenine into quinolinic acid, resulting in the excessive accumulation of glutamate and inhibition of brain-derived neurotrophic factor (BDNF) synthesis. These processes ultimately affect neuronal plasticity and integrity, leading to the manifestation of depressive symptoms (8). Hence, targeting the reduction of inflammation in MDD patients holds promise as a potential treatment strategy.

Lots of drugs have been identified for their antidepressant effects mediated by anti-inflammatory mechanisms. Nonsteroidal anti-inflammatory drugs (NSAIDs) inhibit prostaglandin E2 (PGE2) synthesis, thereby suppressing indoleamine 2,3-dioxygenase (IDO) activation and reducing tryptophan conversion to kynurenine, leading to antidepressant effects (9). Omega-3 fatty acids (10), pioglitazone (11), statins (12), and monoclonal antibodies (13) reduce cytokine-induced neurogenesis and apoptosis, consequently diminishing central nervous system inflammation. Minocycline can inhibit the activation of microglial cells, thereby suppressing the inflammatory state of the body (14). N-acetylcysteine (NAC) supplements enhance brain antioxidant defense, decrease pro-inflammatory cytokines in specific brain regions, regulate glutamate levels, facilitate neurogenesis, and reduce apoptosis, all contributing to antidepressant effects (15–17). Corticosteroids modulate the hypothalamic-pituitary-adrenal axis and individual immune regulation, achieving antidepressant effects through their anti-inflammatory properties (18).

Numerous prior meta-analyses have suggested the efficacy and acceptability of anti-inflammatory agents in the treatment of depression. Köhler-Forsberg (19) et al. conducted a study that demonstrated the potential of various anti-inflammatory agents, including NSAIDs, cytokine inhibitors, glucocorticoids, statins, and minocycline, in the treatment of depression or depressive symptoms. However, their analysis was based on a relatively small sample size of only 12 randomized controlled trials (RCTs). The use of different scales to assess the severity of depression in the included RCTs might result in spurious effect sizes in the meta-analysis results. Bai (20) et al. also suggested that anti-inflammatory agents, including NSAIDs, Omega-3 fatty acids, statins, minocycline, and modafinil, exhibit antidepressant effects in both monotherapy and adjunctive treatment settings. However, it should be noted that approximately 26% of the included RCTs involved participants with various comorbidities, such as human immunodeficiency virus (HIV) infection, multiple sclerosis, and diabetes, which could potentially overestimate the antidepressant effect of these drugs. A similar issue can be observed in another network meta-analysis aiming to provide evidence for the optimal MDD treatment involving different anti-inflammatory agents (21). The limitations mentioned above have hindered the effectiveness of the evidence obtained from the aforementioned meta-analyses in guiding clinical practice.

Therefore, we conducted a meta-analysis to assess the efficacy and acceptability of anti-inflammatory agents in treating MDD patients without any comorbidities. Additionally, a network meta-analysis was performed to determine the optimal treatment among various anti-inflammatory agents, including NSAIDs, corticosteroids, monoclonal antibodies, statins, pioglitazone, minocycline, N-acetylcysteines (NACs) and omega-3 fatty acids.

## Materials and methods

### Search strategy and selection criteria

We conducted both traditional pairwise meta-analysis and network meta-analysis by systematically searching PubMed, Embase, Web of Science, Cochrane review, and Cochrane Trial from their inception to January 26, 2023, with language restrictions set to English. The search targeted previously published articles incorporating the terms “depression disorder” and the specified anti-inflammatory agents mentioned above (**Supplementary Table S1**). We specifically sought double-blind, randomized controlled trials (RCTs) comparing anti-inflammatory agents with a placebo for the treatment of acute depression in adults of both sexes aged 18 years and older. These trials could involve either anti-inflammatory drugs alone (anti-inflammatory agent vs. placebo) or in combination with antidepressant drugs (anti-inflammatory agent + antidepressant drug vs. placebo + antidepressant drug). Inclusion criteria required studies to use established diagnostic criteria for identifying patients with major depressive disorder, such as Feighner criteria, any version of DSM, and ICD-10. We excluded incomplete trials, those involving participants with psychotic depression or seasonal depression, as well as trials with participants having severe endocrine, metabolic, or other diseases. Additionally, trials were excluded if 20% or more of participants had bipolar disorder. For a comprehensive search, we utilized ClinicalTrials.gov, using the following strategies: “studies with results”, “interventional studies”, “depression disorder”, and “anti-inflammatory agents”.

### Outcome assessment and data extraction

Our primary outcomes comprise efficacy and acceptability. Efficacy is measured by the response rate, indicating patients with a ≥50% reduction in the total score of standardized depression assessment scales. Acceptability is measured by the rate of patient dropouts due to all-causes. Secondary outcomes include remission rate, defined as MADRS ≤ 7, HAMD ≤ 7, GDS ≤ 11, or BDI-II ≤ 8 at the end of the trial, and the proportion of patients who dropped out due to adverse events (AE) (**Supplementary Table S2**).

To retrieve relevant studies, Min Wang and Yushun Yan imported all retrieved studies into Endnote and removed duplicate studies. Then, Yue Du and Yikai Dou independently screened the titles and abstracts of each article and reviewed the full text based on our inclusion and exclusion criteria. In case of disagreement, Xiaohong Ma and Xiao Yang jointly reviewed and made the final decision. Huanhuan Fan and Ningdan Fan recorded study information, including author name, publication date, sample sizes, and patient characteristics such as age and gender. In addition, Xiao Yang recorded intervention details such as intervention classification, treatment duration, treatment efficacy, and other clinical outcomes.

### Data analysis

We used Review Manager 5.3 for traditional pairwise meta-analysis to assess the efficacy and acceptability of anti-inflammatory agents. For network meta-analysis, we used a frequentist framework model in Stata (version 17) software. As all our results are binary variables, we calculated odds ratios (ORs) and 95% confidence intervals (CIs) to present the findings. Random-effects models were used for both traditional pairwise meta-analysis and network meta-analysis. Additionally, we conducted subgroup meta-analysis based on different inflammatory drugs and other feathers. To rank the efficacy and acceptability of different anti-inflammatory drugs, we used the surface under the cumulative ranking curve (SUCRA) values. Heterogeneity for each anti-inflammatory drug was quantified using the I^2^ statistic and *p*-value *(* 22). According to the Cochrane Handbook, heterogeneity values of 0–40% were deemed insignificant, 30–60% indicated moderate heterogeneity, 50–90% suggested essential heterogeneity, and 75–100% represented appreciable heterogeneity (23). The included RCTs were evaluated by using the Cochrane Risk of Bias (RoB 2) tool, version 2 (24), which includes five domains for assessing bias such as randomization process, deviations from intended interventions, missing outcome data, measurement of outcomes, and selection of the reported results. When assessing whether outcome data for all participants were completed, we set the cut-off value at 80% (25). Leave-one-out sensitivity analyses were performed, and meta-regression was conducted to adjust for the effect of publication year and treatment duration. To investigate published bias, we used comparison-adjusted funnel plots. Egger’s test was also conducted to test the published bias. We performed the trim and fill procedure to further assess the possible effect of publication bias in pairwise meta-analysis.

This study is registered with PROSPERO, number CRD42023422004, and was conducted in compliance with the Preferred Reporting Items for Systematic Reviews and Meta-Analyses (PRISMA) extension statement (**Supplementary Table S3**).

## Results

### Characteristics of the included studies

**Figure 1:**
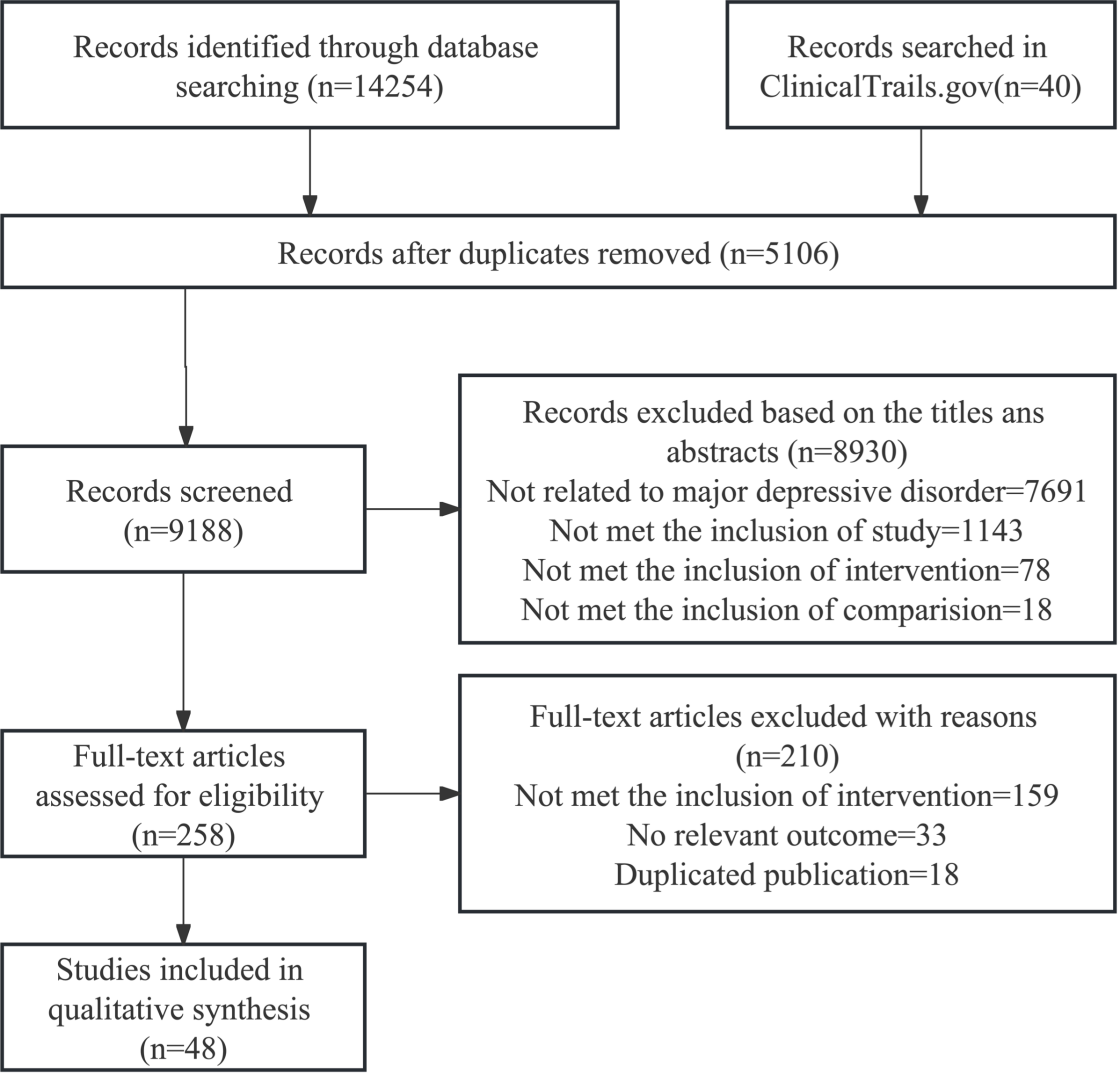
Literature search and selection.

We initially retrieved a total of 14,254 relevant studies. After removing duplicate studies and screening titles/abstracts, we assessed the full text of 258 studies that potentially met the criteria. Finally, 48 randomized clinical trials were included for subsequent data analysis (26–73). The entire process of literature search and trial selection is shown in **Figure 1**. The characteristics of the included trials are summarized in **Supplementary Table S4**. The included studies were published between 1995 and 2022, and involved 3,394 participants, with 53.1% of participants being female. All these trails were placebo-controlled, and the 48 randomized clinical trials employed different interventions, including omega-3 fatty acids (n=19), nonsteroidal anti-inflammatory drugs (n=9), pioglitazone (n=3), minocycline (n=5), NACs (n=2), corticosteroids (n=4), statins (n=4), and monoclonal antibodies (n=2). Sample sizes ranged from 20 to 432, and study durations ranged from 2 days to 16 weeks. In all studies, twenty-eight (56.3%) trials reported efficacy (response rate), including omega-3 fatty acids (n=11), NSAIDs (n=5), pioglitazone (n=1), minocycline (n=4), NACs (n=1), corticosteroids (n=1), statins (n=3), and monoclonal antibodies (n=2), and 45 (93.8%) trials included acceptability of anti-inflammatory agents, including omega-3 fatty acids (n=18), NSAIDs (n=8), pioglitazone (n=3), minocycline (n=4), NACs (n=2), corticosteroids (n=3), statins (n=3), and monoclonal antibodies (n=2). Among all involved RCTs, ten trails were monotherapy (40, 41, 44, 47, 51, 53, 60, 68, 71, 72) and 38 RCTs were adjunctive therapy. Five RCTs focused on treatment-resistant depression (TRD) patient (28, 30, 34, 39, 53). Four RCTs only included female participants (35, 40, 47, 72). Two RCTs included overweight participants (Body Mass Index: BMI ≥ 25 kg/m^2^) (47, 68) and two studies included patients with low-grade inflammation (30, 68). One RCT included participants with preclinically metabolic dysfunction (56) and another included 9 (15%) bipolar disorder patients for exploratory purpose (53).

### Risk of bias

Among all studies, two trials (4.2%) showed a high risk of bias due to inadequate information regarding outcome measurement, and one (2.1%) trial might have a high risk of bias due to missing outcome data. Moreover, twenty trials (41.7%) raised some concerns, while 26 (54.2%) had a low risk. A summary assessment of the risk of bias is illustrated in **Supplementary Figure S1**.

### Pairwise meta-analysis

**Figure 2:**
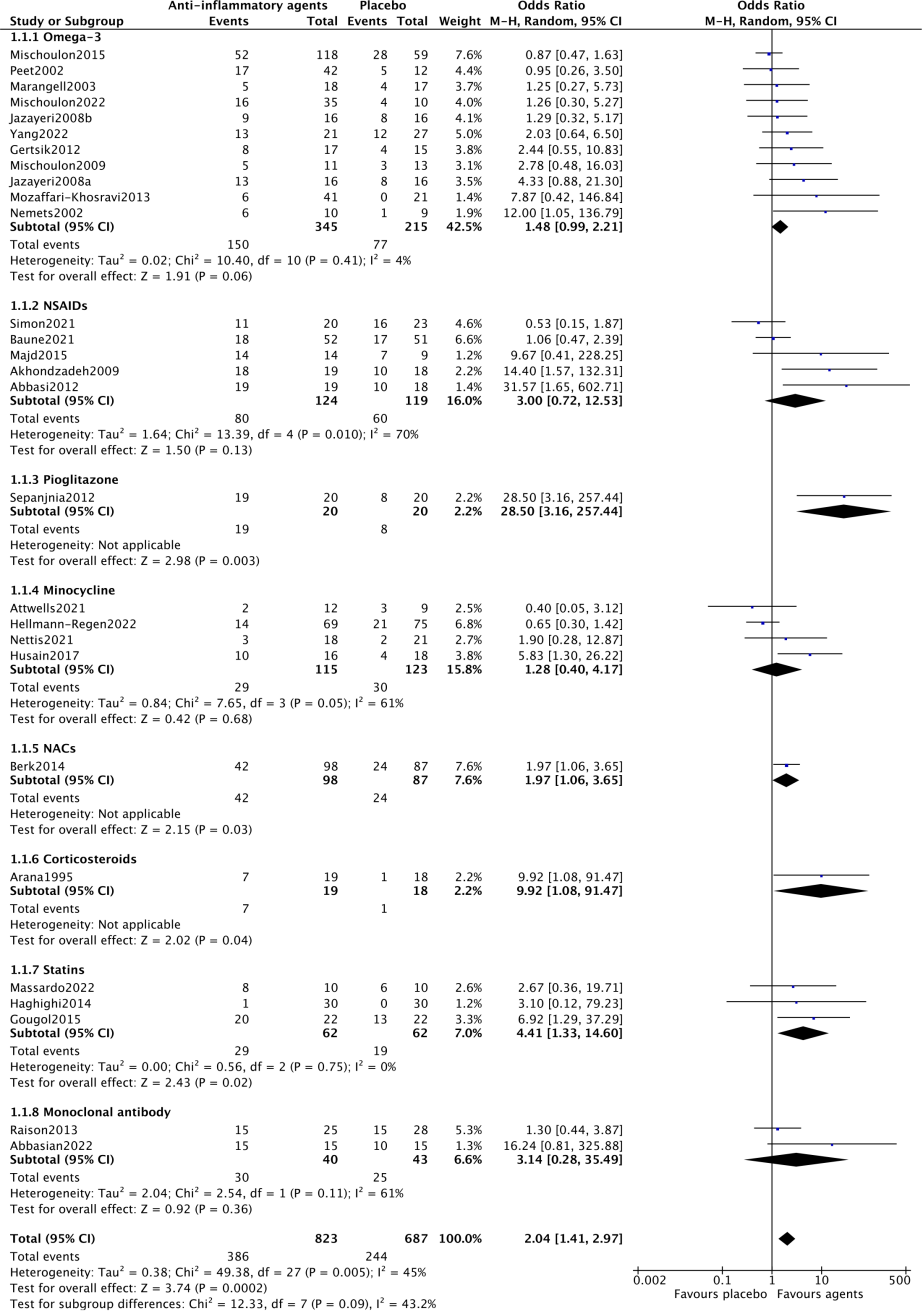
The forest plot of pairwise meta-analysis of efficacy.

Among the included trials, 28 reported efficacy, reflecting the response rate of participants and the efficacy of anti-inflammatory drugs for MDD patients. A random-effects model was used, as shown in **Figure 2**, showing a significant antidepressant effect of anti-inflammatory drugs when compared to placebo (OR=2.04, 95% CI: 1.41–2.97, p=0.0002). However, there was moderate heterogeneity among the trials (Chi^2^ = 49.38, df=27, p=0.005, I^2^ = 45%).

**Figure 3:**
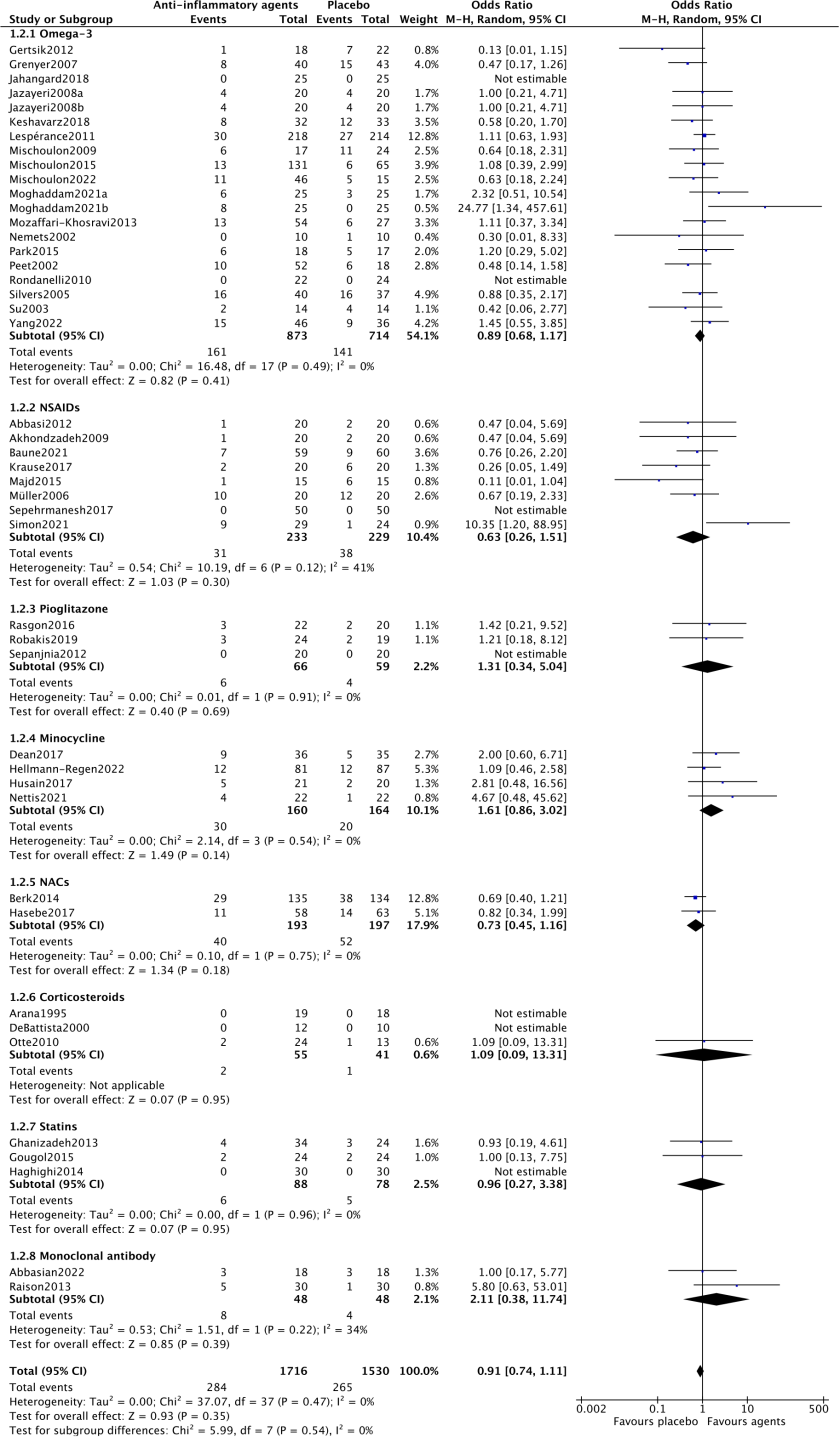
The forest plot of pairwise meta-analysis of acceptability.

For acceptability, forty-three trials assessed the acceptability of anti-inflammatory agents in MDD patients. As shown in **Figure 3**, there was no statistically significant difference in acceptability between anti-inflammatory drugs and placebo (OR=0.91, 95% CI: 0.74–1.11, p=0.35). There was no heterogeneity among the trials (Chi^2^ = 37.07, df=37, p=0.4, I^2^ = 0%).

Twenty-one studies reported remission rates, showing the remission rates of 7 anti-inflammatory drugs in participants. As shown in **Supplementary Figure S2A**, compared with placebo, the remission rate of MDD patients was higher after treatment with anti-inflammatory drugs (OR=1.89, 95% CI: 1.20–2.98, p=0.006). There was relatively low heterogeneity among the studies (Chi^2^ = 30.82, df=19, p=0.04, I^2^ = 38%).

Sixty studies reported dropout rates due to adverse events (AE). As shown in **Supplementary Figure S2B**, there was no significant difference in dropout rates between anti-inflammatory agents and placebo (OR=1.11, 95% CI: 0.66–1.87, p=0.68). There was no heterogeneity among the studies (Chi^2^ = 12.74, df=15, p=0.62, I^2^ = 0%).

### Subgroup analysis

In the subgroup analysis of different anti-inflammatory agents NACs (OR=1.97, 95% CI: 1.06–3.65, p=0.03) and statins (OR=4.41, 95% CI: 1.33–14.60, p=0.02) showed significantly antidepressant effects. Trials of NSAIDs (Chi^2^ = 13.39, df=4, p=0.01, I^2^ = 70%), minocycline (Chi^2^ = 7.65, df=3, p=0.05, I^2^ = 61%) and monoclonal antibodies (Chi^2^ = 2.54, df=1, p=0.11, I^2^ = 61%) showed essential heterogeneity (**Figure 2**). All anti-inflammatory agents demonstrated the same acceptability as placebo (**Figure 3**).

In the subgroup analysis of studies with different therapies (monotherapy or adjunctive therapy), different results were observed. In the adjunctive therapy group, anti-inflammatory agents showed a significantly antidepressant effect compared to placebo (OR=2.17, 95% CI: 1.39–3.37, p=0.0006). In the monotherapy group, anti-inflammatory agents didn’t demonstrate a better antidepressant effect compared to placebo (OR=1.71, 95% CI: 0.88–3.31, p=0.11). Trials of adjunctive therapy (Chi^2^ = 46.04, df=22, p=0.002, I^2^ = 52%) showed moderate heterogeneity, but trials of monotherapy (Chi^2^ = 3.32, df=4, p=0.51, I^2^ = 0%) showed insignificant heterogeneity (**Supplementary Figure S3A**). Both therapies showed the same acceptability as placebo (**Supplementary Figure S3B**).

In the subgroup analysis of studies included treatment-resistant depression patients, anti-inflammatory agents in TRD group showed the same antidepression effect as placebo (OR=0.54, 95% CI: 1.25–2.89, p=0.60). However, in studies only including MDD patients, anti-inflammatory agents demonstrated a better antidepression effect compared to placebo (OR=2.33, 95% CI: 1.53–3.54, p<0.0001). Trials including TRD patients (Chi^2^ = 7.79, df=4, p=0.10, I^2^ = 49%) showed moderate heterogeneity, but trials including MDD group (Chi^2^ = 38.93, df=22, p=0.01, I^2^ = 43%) also showed moderate heterogeneity (**Supplementary Figure S4A**). Both kinds of studies showed the same acceptability as placebo (**Supplementary Figure S4B**).

In the subgroup analysis for mixed sexes, anti-inflammatory agents in mixed sexes group showed a better antidepression effect compared to placebo (OR=2.00, 95% CI: 1.37–2.92, p=0.0003). Trials including mixed sexes (Chi^2^ = 48.11, df=26, p=0.005, I^2^ = 46%) showed moderate heterogeneity (**Supplementary Figure S5A**). The subgroup analysis for both sex types demonstrated similar acceptability to placebo (**Supplementary Figure S5B**). Only one RCTs, which included only female patients, reported the efficacy.

In the subgroup analysis of studies without limitation of BMI, anti-inflammatory agents in showed a better antidepression effect compared to placebo (OR=2.11, 95% CI: 1.43–3.11, p=0.0002). Trials without BMI limitation (Chi^2^ = 49.27, df=26, p=0.004, I^2^ = 47%) showed moderate heterogeneity (**Supplementary Figure S6A**). Studies without limitation of BMI showed the same acceptability as placebo (**Supplementary Figure S6B**). Only one RCTs, which included overweight patients, reported the efficacy.

In the subgroup analysis for studies without inflammatory patients, anti-inflammatory agents showed a better antidepression effect compared to placebo (OR=2.13, 95% CI: 1.43–3.18, p=0.0002) and exhibited moderate heterogeneity (Chi^2^ = 49.26, df=25, p=0.003, I^2^ = 49%) (**Supplementary Figure S7A**). The trials that excluded patients with low-grade inflammation demonstrated comparable acceptability to placebo (**Supplementary Figure S7B**).

In the subgroup analysis for studies without preclinically metabolic dysfunction patients, anti-inflammatory agents showed a better antidepression effect compared to placebo (OR=2.13, 95% CI: 1.43–3.18, p=0.0002) and exhibited moderate heterogeneity (Chi^2^ = 49.38, df=27, p=0.005, I^2^ = 45%) (**Supplementary Figure S8A**). The trials that excluded patients with preclinically metabolic dysfunction demonstrated comparable acceptability to placebo (**Supplementary Figure S8B**).

In the subgroup analysis for studies without bipolar disorder participants, anti-inflammatory agents showed a better antidepression effect compared to placebo (OR=2.12, 95% CI: 1.43–3.15, p=0.0002) and exhibited moderate heterogeneity (Chi^2^ = 49.24, df=26, p=0.004, I^2^ = 47%) (**Supplementary Figure S9A**). The trials that excluded bipolar disorder patients demonstrated comparable acceptability to placebo (**Supplementary Figure S9B**).

Excluding high-risk studies, anti-inflammatory agents still showed a better antidepression effect compared to placebo (OR=2.04, 95% CI: 1.41–2.97, p=0.0002) and exhibited moderate heterogeneity (Chi^2^ = 49.38, df=27, p=0.005, I^2^ = 45%) (**Supplementary Figure S10A**). The trials excluding high risk studies still demonstrated comparable acceptability to placebo (**Supplementary Figure S10B**).

### Network meta-analysis

**Supplementary Figure S11** show the comparison network diagrams between different interventions. However, we found that in the included studies, no trial directly compared two anti-inflammatory drugs, and all comparisons were between anti-inflammatory agents and placebo. Therefore, network meta-analysis was used to conduct direct and indirect comparisons of anti-inflammatory drugs. Each network plot did not form a closed loop, so we did not test for inconsistency in the NMA and only selected the consistency model.

**Supplementary Table S5** shows the results of efficacy and acceptability in the NMA. For efficacy (response rate), statins (OR=4.27, 95% CI: 1.04–17.56) were identified as more efficacious than placebo. Regarding acceptability (all-cause dropout), all anti-inflammatory drugs were found to be as acceptable as the placebo. Notably, more patients dropped out for all causes in minocycline trials compared to NSAIDs (OR=0.41, 95% CI: 0.17–0.98) and NACs (OR=0.45, 95% CI: 0.20–0.99). SUCRAs and cumulative probability plots are presented in **Supplementary Table S6**.

### Sensitivity analysis and meta-regression

The sensitivity analysis results also confirmed the stability of the analytical outcome, indicating that the results remained consistent when employing the leave-one-out method (**Supplementary Figure S12**). Additionally, meta-regression analysis showed no significant correlations between publication year (*p*=0.08) and duration of treatment (*p*=0.43).

### Publication bias

**Figure 4:**
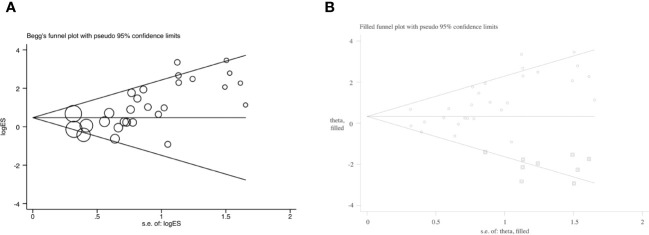
**(A)** Begg’s funnel plot of efficacy; **(B)** Funnel plot of efficacy with trim and fill.

The funnel plot displayed an asymmetrical funnel shape (**Supplementary Figure S13**). The Egger test (*p*<0.001) and Begg test (p=0.001) for studies reported efficacy also raised concerns about potential publication bias (**Figure 4A**). To explore this further, a trim and fill method was employed. The analysis resulted in a changed outcome, which indicated the instability of the outcome with publication bias (OR=1.39, 95% CI: 0.93–2.09) (**Figure 4B**). It’s noteworthy that studies reporting acceptability didn’t exhibit publication bias (Egger test: *p*=0.48; Begg test: *p*=0.63).

In subgroup analysis for adjunctive therapy, both the Egger test (*p*=0.001) and Begg test (*p*=0.008) for studies reporting efficacy suggested a high possibility of publication bias, and the outcome of the meta-analysis was changed in trim and fill analysis (OR=1.39, 95% CI: 0.87–2.21). However, monotherapy group didn’t show publication bias (Egger test: *p*=0.07; Begg test: *p*=0.03).

Similarly, in the subgroup analysis for studies including MDD patients, both the Egger test (*p*<0.001) and Begg test (*p*=0.001) for studies reporting efficacy suggested a high possibility of publication bias, and the outcome of the meta-analysis was changed in trim and fill analysis (OR=1.44, 95% CI: 0.91–2.28). However, studies included TRD patients didn’t show publication bias (Egger test: *p*=0.49; Begg test: *p*=0.81).

## Discussion

The meta-analysis of 28 RCTs indicated a significant antidepressant effect of anti-inflammatory agents compared to placebo in patients with MDD. The treatment effect estimates from our study align with previous reviews on the same topic, but they are significantly more precise due to our larger dataset of 3394 participants and only inclusion of purely diagnosed patients. Subgroup analysis further revealed that specific agents such as NACs and statins exhibited significant antidepressant effects. Both adjunctive therapy group and the MDD patients’ group showed a significant antidepressant effect with anti-inflammatory agents compared to placebo. In terms of acceptability, the meta-analysis of 43 RCTs demonstrated that anti-inflammatory agents were comparable to placebo. Notably, among the anti-inflammatory agents analyzed through network meta-analysis, NSAIDs displayed the highest level of acceptability, although its efficacy is comparable to placebo.

Our primary findings regarding the efficacy of anti-inflammatory agents align with previous meta-analyses, and these results remain consistent regardless of treatment duration or publication year (19–21). However, the identification of significant publication bias underlines the need for caution in interpreting the overall results. The bias may be attributed to an abundance of small-scale studies. In subgroup analysis, NACs and statins demonstrated a noteworthy antidepressant effect. It is worth noting that only one RCT was included in NACs subgroups. In our meta-analysis, there was no significant difference in the antidepressant efficacy between NSAIDs and minocycline compared to placebo. The existing studies on the antidepressant effects of NSAIDs have yielded inconsistent findings. While some studies suggested that NSAIDs could alleviate depression symptoms by reducing inflammation (38, 73), the antidepressant effect of NSAIDs may be influenced by the participants’ inflammatory levels (27, 42) and the potential interaction between NSAIDs and antidepressant medications (74). It appears that NSAIDs may struggle to exert antidepressant effects in the treatment of MDD patients without elevated inflammation. On the other hand, as one of the most commonly prescribed medications globally, NSAIDs have consistently shown good acceptability (75). Therefore, NSAIDs may hold promise for clinical application in MDD patients, particularly in cases where inflammatory levels are significantly elevated. The RCTs examining minocycline solely focused on treatment-resistant depression (TRD) patients, and the adjunctive use of minocycline did not significantly alleviate depression symptoms in this meta-analysis. This finding contrasts with previous meta-analyses suggesting that minocycline has an antidepressant effect. However, it is important to note that those earlier meta-analyses included RCTs involving MDD patients with HIV infection (76) and secondary progressive multiple sclerosis (77), and the scales used to measure depressive severity were not uniform (26). These factors could potentially impact the results of the meta-analyses.

Additionally, the efficacy analysis revealed contrasting outcomes between the adjunctive therapy group and the monotherapy group. While monotherapy with anti-inflammatory agents failed to exhibit superior antidepressant effects compared to placebo, adjunctive therapy demonstrated a significant antidepressant effect, consistent with the trend observed in previous meta-analysis (20). This discrepancy may arise from the fact that anti-inflammatory agents accelerate the reduction of depressive symptoms when combined with antidepressant drugs during early treatment phases, but do not independently exert antidepressant effects (35, 42). Our meta-analysis suggested that inadequate evidence supporting the antidepressant effect of anti-inflammatory agents in treatment-resistant depression (TRD) patients. But we included only five randomized controlled trials (RCTs) with relatively small sample sizes (total participants in four out of five RCTs ≤53). Therefore, further studies are necessary to provide a more comprehensive analysis of this topic.

In our network meta-analysis, statins demonstrated greater efficacy than placebo in the treatment of patients with MDD. The antidepressant effect of statins aligns with previous meta-analyses (78). Animal experiments have also suggested that statins can reduce depressive-like behaviors in mice and rats by suppressing microglial and astrocyte activation, as well as cytokine release in the central nervous system. This inhibition occurs through the nuclear factor-kB pathway, thereby reducing the secretion of IL-1B, IL-6, and TNF-α (79–81).

Significant publication bias is worth noting in this meta-analysis, likely due to the limited sample sizes and insufficient number of RCTs for each anti-inflammatory agent. Consequently, further studies are needed to investigate the antidepressant effects of anti-inflammatory agents.

Several limitations exist in our study. Firstly, we focused on widely reported anti-inflammatory agents with potential antidepressant efficacy, leaving other anti-inflammatory drugs with potential antidepressant effects unexplored. Secondly, we have not considered the dosage of the drugs in our analysis, making it difficult to determine the optimal dosage for achieving antidepressant efficacy. Thirdly, the results of depressive scales were not analyzed, which could have provided additional information regarding the antidepressant effects of the included anti-inflammatory agents.

## Conclusion

In conclusion, anti-inflammatory agents, overall, demonstrate significant effectiveness in treating major depressive disorder compared to placebo, while maintaining comparable acceptability. Subgroup analysis revealed that NACs and statins also exhibited significant antidepressant effects compared to placebo. Moreover, both adjunctive therapy and exclusively MDD patients’ groups showed a significant antidepressant effect with anti-inflammatory agents when compared to placebo. In the network meta-analysis, NSAIDs displayed the highest level of acceptability. To address significant publication bias, further high-quality RCTs with larger sample sizes, MDD patients without comorbidities, and consistent depression severity are crucial. These future trials will contribute to a stronger evidence base in this field.

## Author contributions

YDU: Writing – review & editing, Writing – original draft. YDO: Writing – review & editing, Writing – original draft. MW: Writing – review & editing, Validation, Data curation. YW: Writing – review & editing, Data curation. YY: Writing – review & editing, Data curation. HF: Writing – review & editing, Visualization, Validation. NF: Writing – review & editing, Validation, Data curation. XY: Writing – review & editing, Project administration, Funding acquisition. XM: Writing – review & editing, Project administration, Funding acquisition.
