# Supplementary material for: Efficacy and acceptability of anti-inflammatory agents in major depressive disorder: a systematic review and meta-analysis
Source: Front Psychiatry. 2024 May 28;15:1407529. doi: 10.3389/fpsyt.2024.1407529 (PMC11165078; doi:10.3389/fpsyt.2024.1407529)
Supplement: Supplementary file 1 [file DataSheet_1.zip › Supplementary Figure 11.DOCX]

Fig. S11. (A) Network map for efficacy. (B)Network map for acceptability.

The width of the line indicates the number of trials comparing two agents. The size of the node indicates the number of MDD patients randomized to a particular agent. NSAIDs: non-steroidal anti-inflammatory drugs; NACs: N-acetylcysteines.

1. Efficacy (Response rate)

1. Acceptability (All-cause dropout rate)
