# Supplementary material for: Efficacy and acceptability of anti-inflammatory agents in major depressive disorder: a systematic review and meta-analysis
Source: Front Psychiatry. 2024 May 28;15:1407529. doi: 10.3389/fpsyt.2024.1407529 (PMC11165078; doi:10.3389/fpsyt.2024.1407529)

Fig. S2: (A) The forest plot of pairwise meta-analysis of remission;(B) The forest plot of pairwise meta-analysis of dropout rate due to adverse events.

(A):


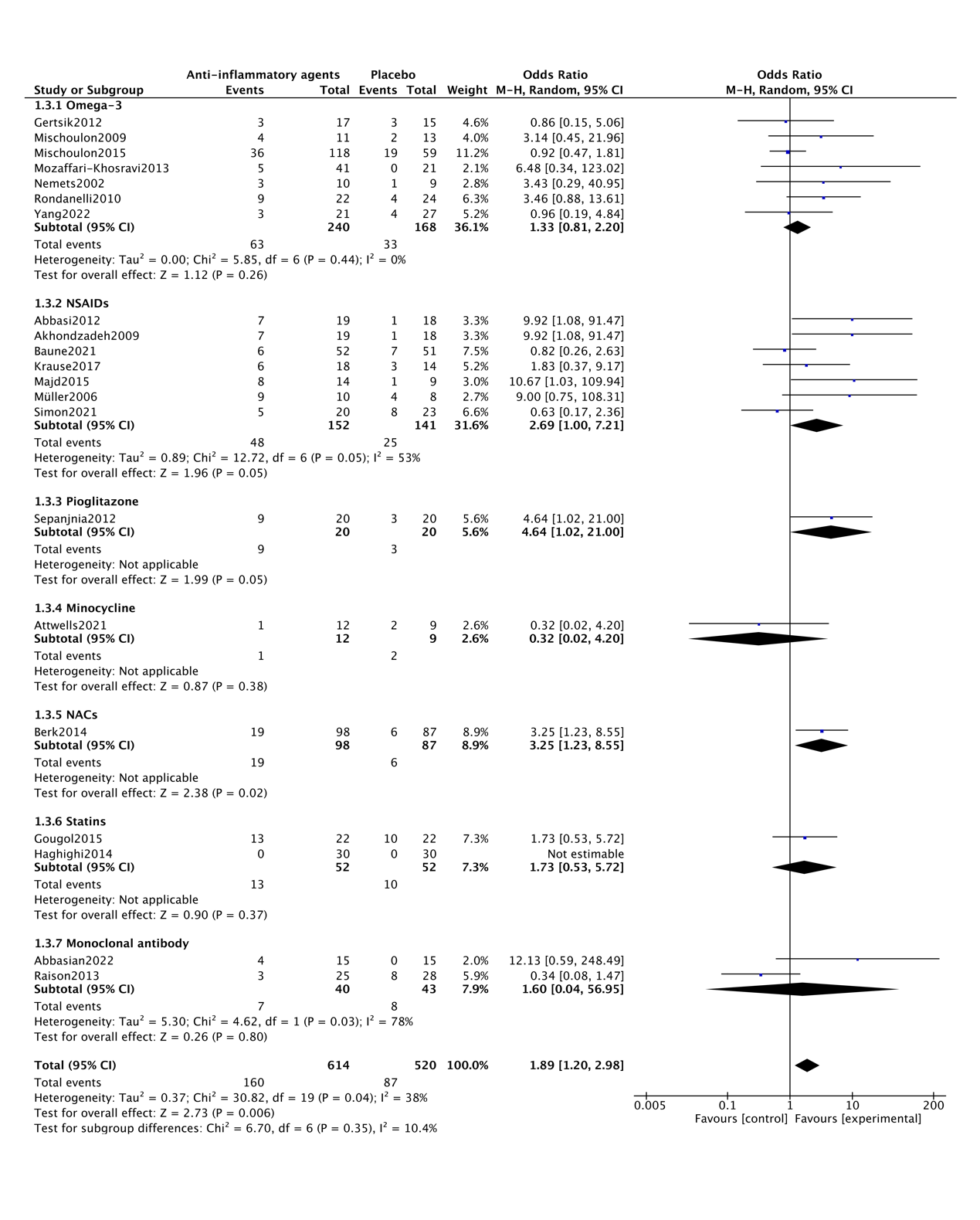


(B):


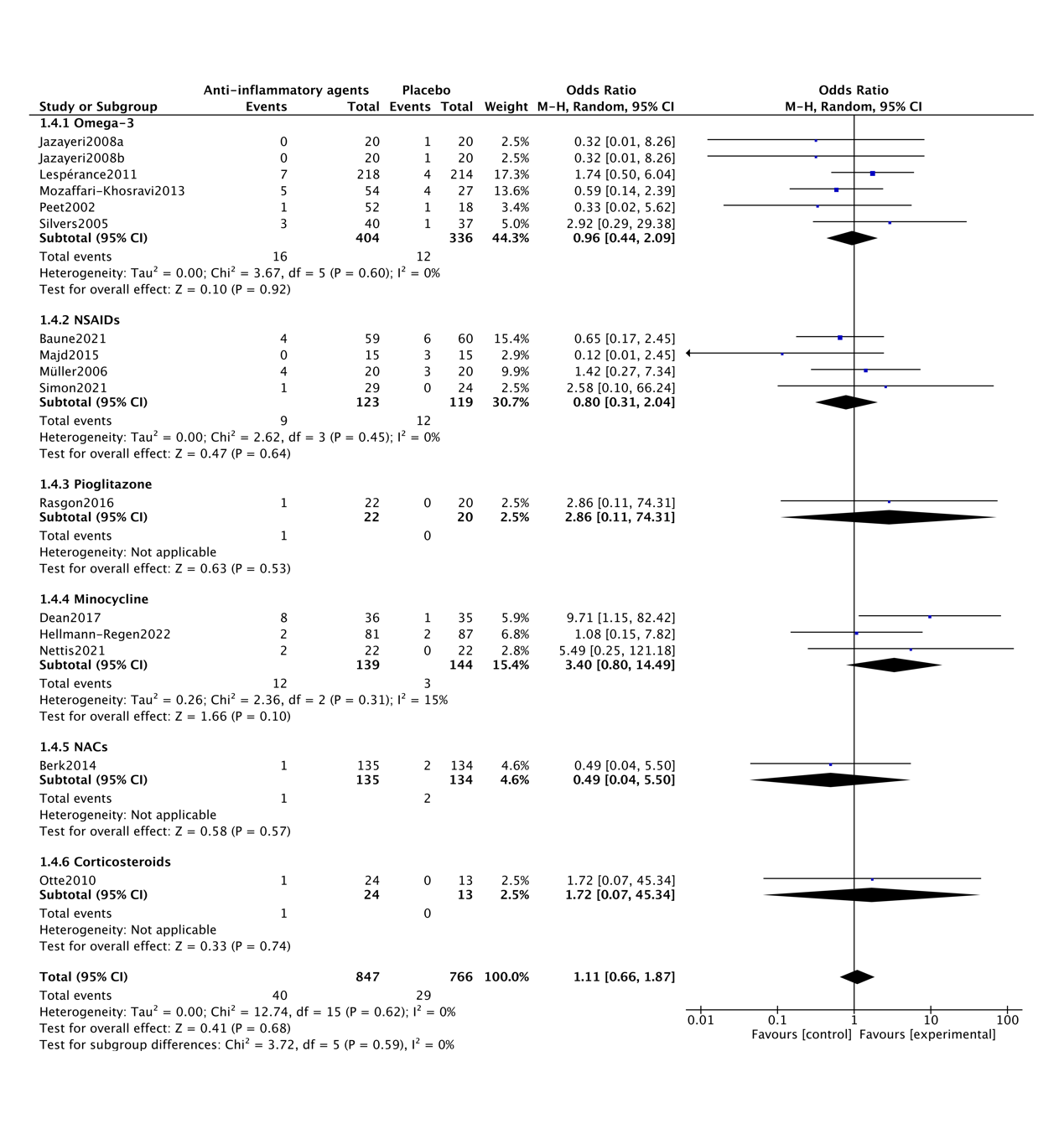

Supplement: Supplementary file 1 [file DataSheet_1.zip › Supplementary Figure 2.DOCX]
