# Supplementary material for: Efficacy and acceptability of anti-inflammatory agents in major depressive disorder: a systematic review and meta-analysis
Source: Front Psychiatry. 2024 May 28;15:1407529. doi: 10.3389/fpsyt.2024.1407529 (PMC11165078; doi:10.3389/fpsyt.2024.1407529)

Fig. S3: (A) The forest plot of efficacy of the different therapy subgroup analysis;(B) The forest plot of acceptability of the different therapy subgroup analysis.

(A):


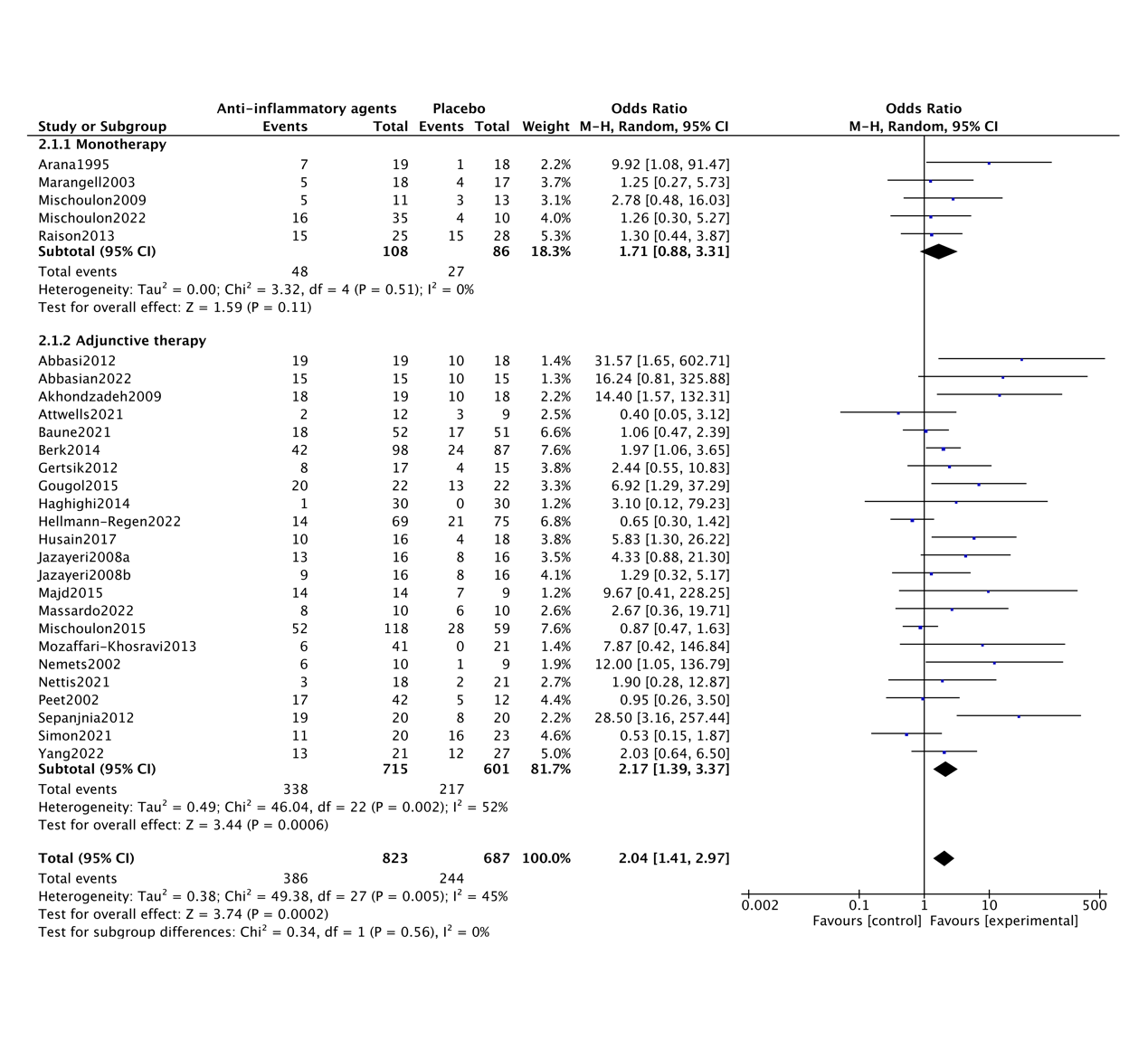


(B):


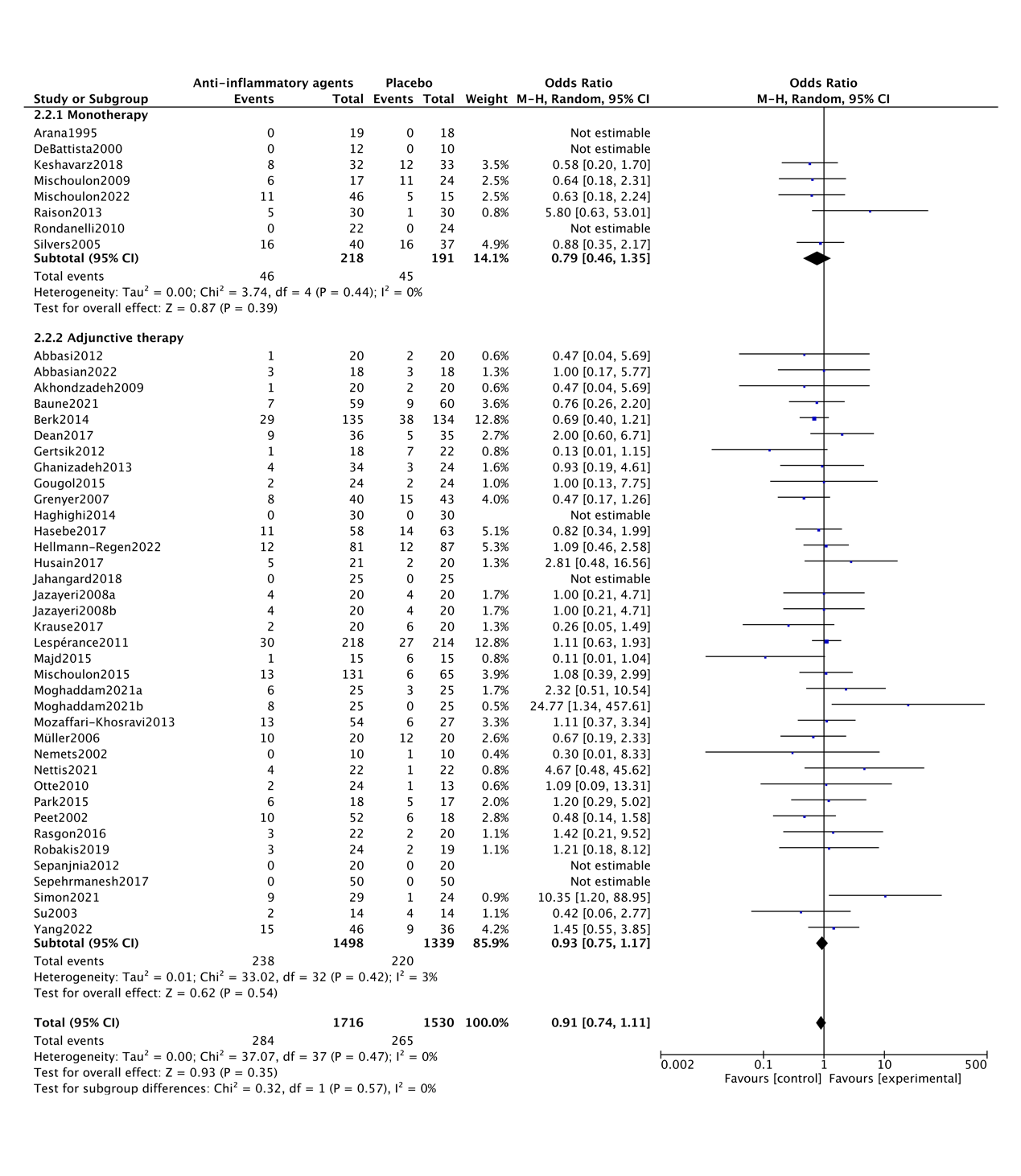


Fig. S4: (A) The forest plot of efficacy of the different patient subgroup analysis;(B) The forest plot of acceptability of the different patient subgroup analysis.

(A):


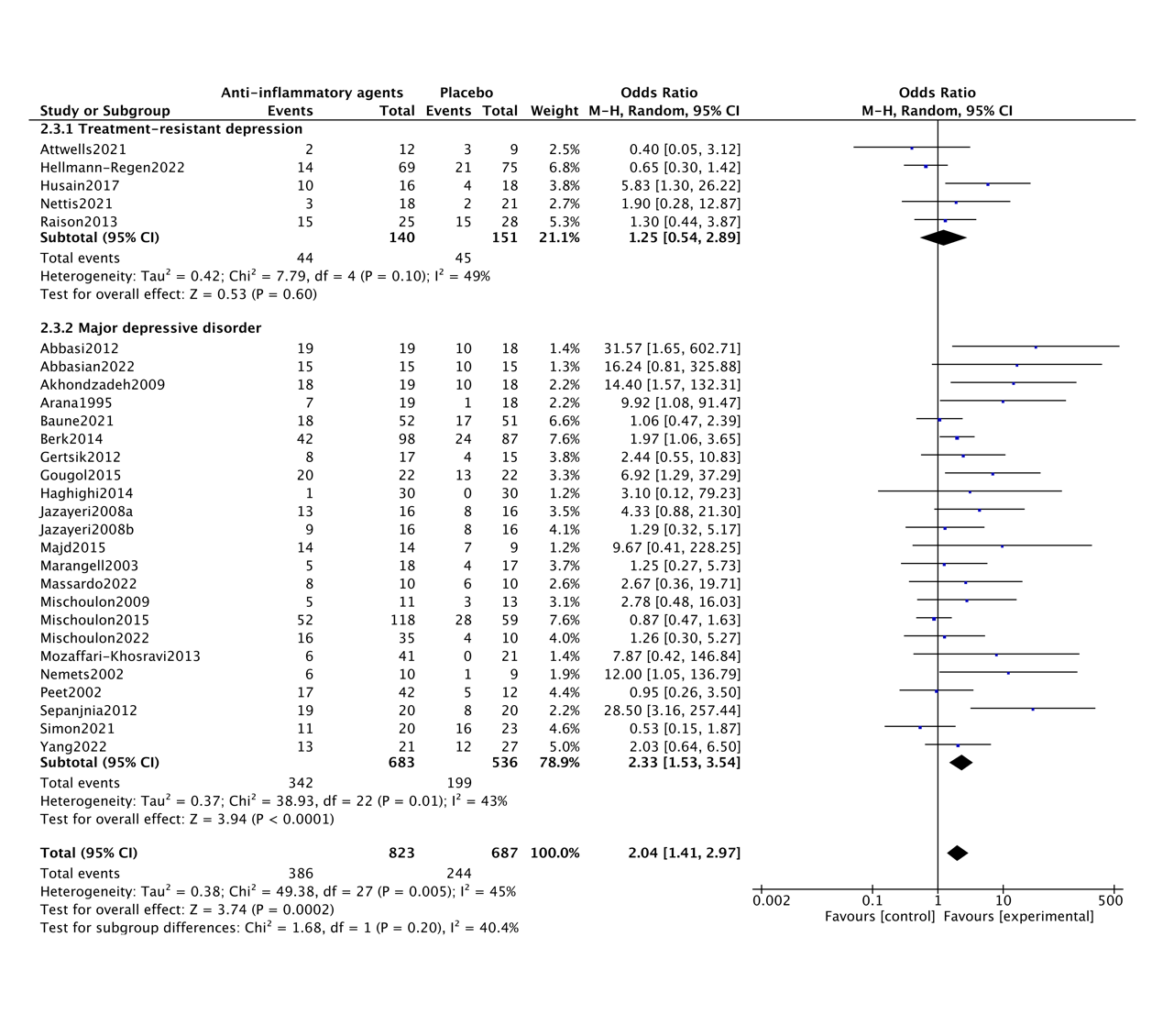


(B):


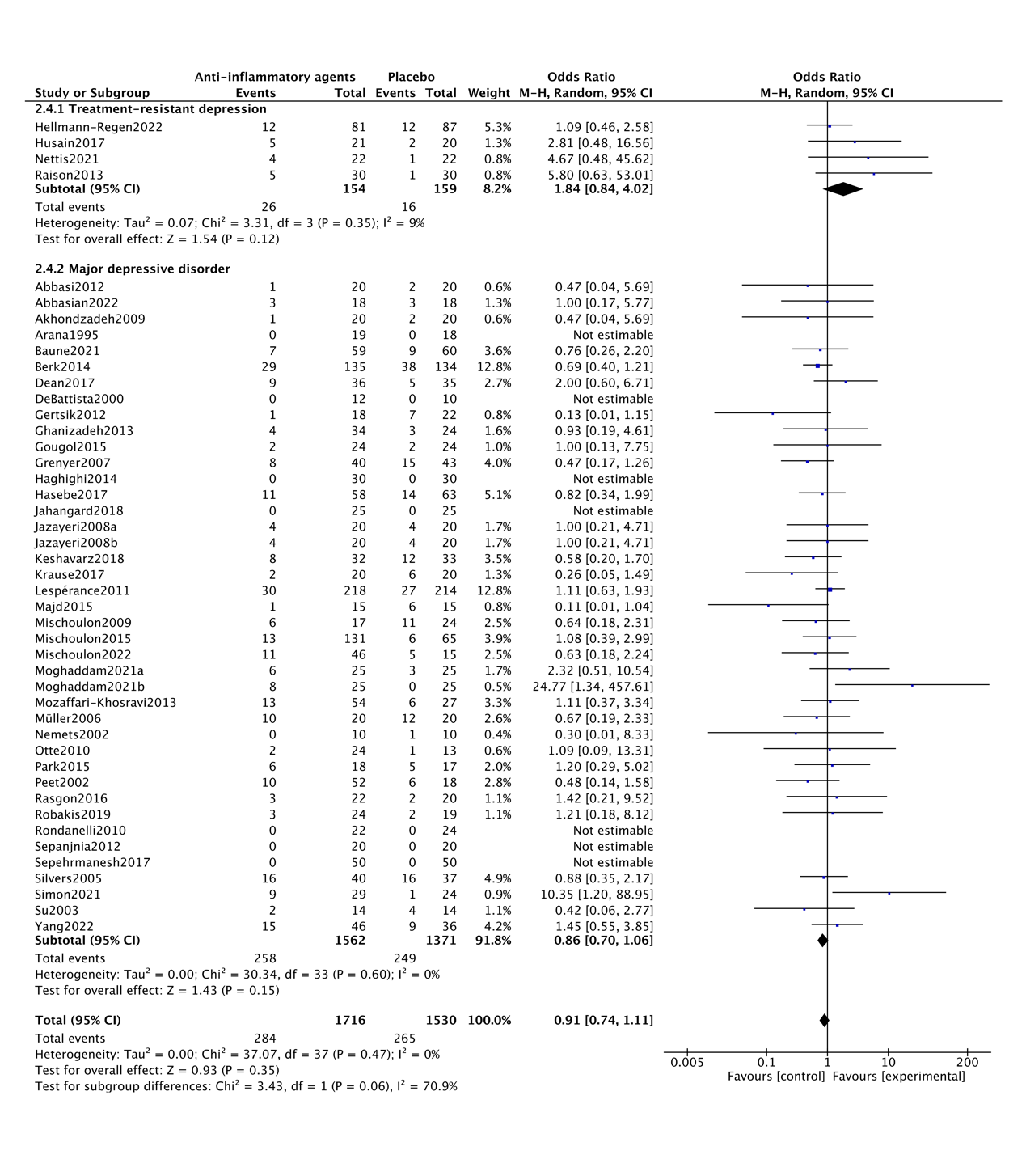

Supplement: Supplementary file 1 [file DataSheet_1.zip › Supplementary Figure 3&4.DOCX]
