# Supplementary material for: Efficacy and acceptability of anti-inflammatory agents in major depressive disorder: a systematic review and meta-analysis
Source: Front Psychiatry. 2024 May 28;15:1407529. doi: 10.3389/fpsyt.2024.1407529 (PMC11165078; doi:10.3389/fpsyt.2024.1407529)
Supplement: Supplementary file 1 [file DataSheet_1.zip › Supplementary Table 1.DOCX]

Table S1 Search strategies

| **Electronic databases** | **Search strategies** |
| --- | --- |
| Cochrane Library and Review | #1 MeSH descriptor: [Anti-Inflammatory Agents] explode all trees  #2 ("Anti‐Inflammatory Agents" OR "Anti Inflammatory Agents" OR "Agents, Antiinflammatory" OR Antiinflammatories OR "Antiinflammatory Agents" OR "Agents, Anti-Inflammatory" OR "Agents, Anti Inflammatory" OR Anti-Inflammatories OR "Anti Inflammatories" OR "Anti-inflammatory agents" OR "Anti-inflammatory drugs" OR "Anti-inflammatory drug"):ti,ab,kw (Word variations have been searched)  #3 MeSH descriptor: [Anti-Inflammatory Agents, Non-Steroidal] explode all trees  #4 ("Anti-Inflammatory Agents, Non-Steroidal" OR nsaid OR "Antiinflammatory Agents, Non Steroidal" OR NSAIDs OR "Non-Steroidal Anti-Inflammatory Agents" OR "Non Steroidal Anti Inflammatory Agents" OR "Nonsteroidal Anti-Inflammatory Agents" OR "Nonsteroidal Anti Inflammatory Agents" OR "Anti Inflammatory Agents, Nonsteroidal" OR "Antiinflammatory Agents, Nonsteroidal" OR "Nonsteroidal Antiinflammatory Agents" OR "Analgesics, Anti-Inflammatory" OR "Anti-Inflammatory Analgesics" OR "Aspirin-Like Agents" OR "Aspirin Like Agents" OR "Cyclooxygenase Inhibitors" OR "Inhibitors, Cyclo-Oxygenase" OR "Inhibitors, Cyclo Oxygenase" OR "Inhibitors, Cyclooxygenase" OR "Prostaglandin Synthesis Antagonists" OR "Antagonists, Prostaglandin Synthesis" OR "Inhibitors, Prostaglandin-Endoperoxide Synthase" OR "Inhibitors, Prostaglandin Endoperoxide Synthase" OR "Prostaglandin Endoperoxide Synthase Inhibitors" OR "Prostaglandin Synthase Inhibitors" OR "Cyclo-Oxygenase Inhibitors" OR "Cyclo Oxygenase Inhibitors" OR "Inhibitors, Prostaglandin Synthase" OR "Cyclooxygenase 2 Inhibitors" OR "Inhibitors, Cyclooxygenase 2" OR "Cyclooxygenase-2 Inhibitors" OR "Inhibitors, Cyclooxygenase-2" OR Coxibs OR "COX-2 Inhibitors" OR "COX 2 Inhibitors" OR "Inhibitors, COX-2" OR "COX2 Inhibitors" OR "Inhibitors, COX2" OR "non‐steroidal anti inflammat*" OR "non‐steroidal anti‐inflammat*" OR cyclooxygenase OR cyclo‐oxygenase OR aspirin OR acetylsalicyl* OR "carbasalate calcium" OR diflunisal OR aceclofenac OR alclofenac OR diclofenac OR ndometacin OR indomethacin OR sulindac OR piroxicam OR dexibuprofen OR dexketoprofen OR fenoprofen OR flurbiprofen OR ibuprofen OR ketoprofen OR naproxen OR tiapro* OR metamizol OR phenylbutazone OR phenazone OR propyphenazone OR etoricoxib OR nabumeton OR parecoxib OR rofecoxib OR celecoxib OR valdecoxib OR lumiracoxib OR vioxx OR celebrex OR bextra OR prexige OR arcoxia OR etodolac OR floctafenine OR meclofenam* OR meloxicam OR oxaprozin OR piroxicam OR tenoxicam OR tolmetin OR acetaminophen OR paracetamol OR rofecoxib):ti,ab,kw (Word variations have been searched)  #5 MeSH descriptor: [Cyclooxygenase Inhibitors] explode all trees  #6 MeSH descriptor: [Cyclooxygenase 2 Inhibitors] explode all trees  #7 MeSH descriptor: [Adrenal Cortex Hormones] explode all trees  #8 MeSH descriptor: [Glucocorticoids] explode all trees  #9 ("Adrenal Cortex Hormones" OR "Hormones, Adrenal Cortex" OR Corticosteroids OR Corticoids OR glucocorticoids OR Glucocorticoid OR "Glucocorticoid Effect" OR "Effect, Glucocorticoid" OR "Glucorticoid Effects" OR "Effects, Glucorticoid" OR corticoid OR adrenocorticoid OR hydrocortisone OR chronocort OR plenadren OR beclomethasone OR betamethasone OR budesonide OR cortisone OR dexamethasone OR methylprednisolone OR prednisolone OR prednisone OR triamcinolone OR meprednisone OR betametasone OR dexametasone):ti,ab,kw (Word variations have been searched)  #10 MeSH descriptor: [Antibodies, Monoclonal] explode all trees  #11 ("cytokine inhibitor" OR "TNF inhibitor" OR "tumour necrosis factor inhibitor" OR "tumor necrosis factor inhibitor infliximab" OR etanercept OR adalimumab OR ustekinumab OR "Antibodies, Monoclonal" OR "Monoclonal Antibodies"):ti,ab,kw (Word variations have been searched)  #12 MeSH descriptor: [Hydroxymethylglutaryl-CoA Reductase Inhibitors] explode all trees  #13 ("Hydroxymethylglutaryl-CoA Reductase Inhibitors" OR "Hydroxymethylglutaryl CoA Reductase Inhibitors" OR "Inhibitors, Hydroxymethylglutaryl-CoA Reductase" OR "Reductase Inhibitors, Hydroxymethylglutaryl-CoA" OR "Inhibitors, HMG-CoA Reductase" OR "Inhibitors, HMG CoA Reductase" OR "Reductase Inhibitors, HMG-CoA" OR "HMG-CoA Reductase Inhibitors" OR "HMG CoA Reductase Inhibitors" OR "Statins, HMG-CoA" OR "HMG-CoA Statins" OR "Statins, HMG CoA" OR "Inhibitors, Hydroxymethylglutaryl-CoA" OR "Hydroxymethylglutaryl-CoA Inhibitors" OR "Inhibitors, Hydroxymethylglutaryl CoA" OR Statins OR "Inhibitors, Hydroxymethylglutaryl-Coenzyme A" OR "Hydroxymethylglutaryl-Coenzyme A Inhibitors" OR "Inhibitors, Hydroxymethylglutaryl Coenzyme A" OR "HMG-CoA reductase inhibitor*" OR "lipid-lowering drug" OR "lipid-lowering agent" OR simvastatin OR pravastatin OR atorvastatin OR rosuvastatin OR Fluvastatin OR Lovastatin OR Pitavastatin OR statin):ti,ab,kw (Word variations have been searched)  #14 MeSH descriptor: [Minocycline] explode all trees  #15 (minocycline OR "Minox 50" OR Aknemin OR Aknin-Mino OR "Aknin Mino" OR Aknosan OR Mynocine OR Apo-Minocycline OR "Apo Minocycline" OR Arestin OR Blemix OR Cyclomin OR Cyclops OR Dentomycin OR Dynacin OR Icht-Oral OR "Icht Oral" OR Klinomycin OR Lederderm OR Mestacine OR Minakne OR Mino-Wolff OR "Mino Wolff" OR Minocin OR "Minocin MR" OR Minoclir OR "Minocycline Hydrochloride" OR "Hydrochloride, Minocycline" OR "Minocycline Monohydrochloride" OR "Monohydrochloride, Minocycline" OR Minolis OR Minomycin OR Minoplus OR Minotab OR Akamin OR Akne-Puren OR "Akne Puren"):ti,ab,kw (Word variations have been searched)  #16 MeSH descriptor: [Pioglitazone] explode all trees  #17 (Pioglitazone OR "U 72107A" OR "U72,107A" OR "U-72107A" OR "U72107A" OR "AD 4833" OR "AD-4833" OR "AD4833" OR "Pioglitazone Hydrochloride" OR Actos):ti,ab,kw (Word variations have been searched)  #18 MeSH descriptor: [Fatty Acids, Omega-3] explode all trees  #19 MeSH descriptor: [Fish Oils] explode all trees  #20 MeSH descriptor: [Docosahexaenoic Acids] explode all trees  #21 MeSH descriptor: [Eicosapentaenoic Acid] explode all trees  #22 ("fatty acids" OR "fatty acids, omega 3" OR "fatty acid" OR fats OR omega‐3 OR "omega‐3 fatty acids" OR PUFA* OR Polyunsaturated OR EFA OR EPA OR "eicosapentaenoic acid*" OR ALA OR ALAs OR "alpha-linolenic acid*" OR "alphalinolenic acid*" OR DHA OR DHAs OR "docosahexaenoic acid*" OR DPA OR "docosapentanoic acid*" OR "n‐3‐fatty‐acid*" OR "flaxseed oil" OR "linseed oil" OR "fish oil*" OR "salmon oil" OR "cod liver oil" OR "mackerel oil" OR "tuna* oil" OR "tuna fish oil" OR "blackcurrant oil" OR "canola oil" OR "rapeseed oil" OR "mustard oil*" OR "walnut oil" OR "wheat germ oil" OR "dental oil*" OR "primrose oil"):ti,ab,kw (Word variations have been searched)  #23 MeSH descriptor: [Acetylcysteine] explode all trees  #24 (Acetylcysteine OR N-Acetyl-L-cysteine OR "N Acetyl L cysteine" OR N-Acetylcysteine OR "N Acetylcysteine" OR NAC):ti,ab,kw (Word variations have been searched)  #25 #1 or #2 or #3 or #4 or #5 or #6 or #7 or #8 or #9 or #10 or #11 or #12 or #13 or #14 or #15 or #16 or #17 or #18 or #19 or #20 or #21 or #22 or #23 or #24  #26 MeSH descriptor: [Depressive Disorder] explode all trees  #27 ("Depressive Disorder" OR "Depressive Disorders" OR "Disorder, Depressive" OR "Disorders, Depressive" OR "Neurosis, Depressive" OR "Depressive Neuroses" OR "Depressive Neurosis" OR "Neuroses, Depressive" OR "Depression, Endogenous" OR "Depressions, Endogenous" OR "Endogenous Depression" OR "Endogenous Depressions" OR "Depressive Syndrome" OR "Depressive Syndromes" OR "Syndrome, Depressive" OR "Syndromes, Depressive" OR "Depression, Neurotic" OR "Depressions, Neurotic" OR "Neurotic Depression" OR "Neurotic Depressions" OR Melancholia OR Melancholies OR "Unipolar Depression" OR "Depression, Unipolar" OR "Depressions, Unipolar" OR "Unipolar Depressions" OR Depression):ti,ab,kw (Word variations have been searched)  #28 ("Randomized Controlled Trials" OR "Randomized Controlled Trial" OR Randomized OR randomised OR RCT OR random* OR allocate OR assign* OR trial):ti,ab,kw (Word variations have been searched)  #29 #26 or #27  #30 #25 and #28 and #29 |
| Embase | #1 'anti‐inflammatory agents':ab,ti OR 'anti inflammatory agents':ab,ti OR 'agents, antiinflammatory':ab,ti OR antiinflammatories:ab,ti OR 'antiinflammatory agents':ab,ti OR 'agents, anti-inflammatory':ab,ti OR 'agents, anti inflammatory':ab,ti OR 'anti inflammatories':ab,ti OR 'anti-inflammatory agents':ab,ti OR 'anti-inflammatory drugs':ab,ti OR 'anti-inflammatory drug':ab,ti OR 'anti-inflammatory agents, non-steroidal':ab,ti OR nsaid:ab,ti OR 'antiinflammatory agents, non steroidal':ab,ti OR nsaids:ab,ti OR 'non-steroidal anti-inflammatory agents':ab,ti OR 'non steroidal anti inflammatory agents':ab,ti OR 'nonsteroidal anti-inflammatory agents':ab,ti OR 'nonsteroidal anti inflammatory agents':ab,ti OR 'anti inflammatory agents, nonsteroidal':ab,ti OR 'antiinflammatory agents, nonsteroidal':ab,ti OR 'nonsteroidal antiinflammatory agents':ab,ti OR 'analgesics, anti-inflammatory':ab,ti OR 'anti-inflammatory analgesics':ab,ti OR 'aspirin-like agents':ab,ti OR 'aspirin like agents':ab,ti OR 'cyclooxygenase inhibitors':ab,ti OR 'inhibitors, cyclo-oxygenase':ab,ti OR 'inhibitors, cyclo oxygenase':ab,ti OR 'inhibitors, cyclooxygenase':ab,ti OR 'prostaglandin synthesis antagonists':ab,ti OR 'antagonists, prostaglandin synthesis':ab,ti OR 'inhibitors, prostaglandin-endoperoxide synthase':ab,ti OR 'inhibitors, prostaglandin endoperoxide synthase':ab,ti OR 'prostaglandin endoperoxide synthase inhibitors':ab,ti OR 'prostaglandin synthase inhibitors':ab,ti OR 'cyclo-oxygenase inhibitors':ab,ti OR 'cyclo oxygenase inhibitors':ab,ti OR 'inhibitors, prostaglandin synthase':ab,ti OR 'cyclooxygenase 2 inhibitors':ab,ti OR 'inhibitors, cyclooxygenase 2':ab,ti OR 'cyclooxygenase-2 inhibitors':ab,ti OR 'inhibitors, cyclooxygenase-2':ab,ti OR coxibs:ab,ti OR 'cox-2 inhibitors':ab,ti OR 'cox 2 inhibitors':ab,ti OR 'inhibitors, cox-2':ab,ti OR 'cox2 inhibitors':ab,ti OR 'inhibitors, cox2':ab,ti OR 'non‐steroidal anti inflammat*':ab,ti OR 'non‐steroidal anti‐inflammat*':ab,ti OR cyclooxygenase:ab,ti OR cyclo‐oxygenase:ab,ti OR aspirin:ab,ti OR acetylsalicyl*:ab,ti OR 'carbasalate calcium':ab,ti OR diflunisal:ab,ti OR aceclofenac:ab,ti OR alclofenac:ab,ti OR diclofenac:ab,ti OR ndometacin:ab,ti OR indomethacin:ab,ti OR sulindac:ab,ti OR dexibuprofen:ab,ti OR dexketoprofen:ab,ti OR fenoprofen:ab,ti OR flurbiprofen:ab,ti OR ibuprofen:ab,ti OR ketoprofen:ab,ti OR naproxen:ab,ti OR tiapro*:ab,ti OR metamizol:ab,ti OR phenylbutazone:ab,ti OR phenazone:ab,ti OR propyphenazone:ab,ti OR etoricoxib:ab,ti OR nabumeton:ab,ti OR parecoxib:ab,ti OR rofecoxib:ab,ti OR celecoxib:ab,ti OR valdecoxib:ab,ti OR lumiracoxib:ab,ti OR vioxx:ab,ti OR celebrex:ab,ti OR bextra:ab,ti OR prexige:ab,ti OR arcoxia:ab,ti OR etodolac:ab,ti OR floctafenine:ab,ti OR meclofenam*:ab,ti OR meloxicam:ab,ti OR oxaprozin:ab,ti OR piroxicam:ab,ti OR tenoxicam:ab,ti OR tolmetin:ab,ti OR acetaminophen:ab,ti OR paracetamol:ab,ti OR rofecoxiband:ab,ti OR 'adrenal cortex hormones':ab,ti OR 'hormones, adrenal cortex':ab,ti OR corticosteroids:ab,ti OR corticoids:ab,ti OR glucocorticoids:ab,ti OR glucocorticoid:ab,ti OR 'glucocorticoid effect':ab,ti OR 'effect, glucocorticoid':ab,ti OR 'glucorticoid effects':ab,ti OR 'effects, glucorticoid':ab,ti OR corticoid:ab,ti OR adrenocorticoid:ab,ti OR hydrocortisone:ab,ti OR chronocort:ab,ti OR plenadren:ab,ti OR beclomethasone:ab,ti OR betamethasone:ab,ti OR budesonide:ab,ti OR cortisone:ab,ti OR dexamethasone:ab,ti OR methylprednisolone:ab,ti OR prednisolone:ab,ti OR prednisone:ab,ti OR triamcinolone:ab,ti OR meprednisone:ab,ti OR betametasone:ab,ti OR dexametasone:ab,ti OR 'cytokine inhibitor':ab,ti OR 'tnf inhibitor':ab,ti OR 'tumour necrosis factor inhibitor':ab,ti OR 'tumor necrosis factor inhibitor infliximab':ab,ti OR etanercept:ab,ti OR adalimumab:ab,ti OR ustekinumab:ab,ti OR 'antibodies, monoclonal':ab,ti OR 'monoclonal antibodies':ab,ti OR 'hydroxymethylglutaryl-coa reductase inhibitors':ab,ti OR 'hydroxymethylglutaryl coa reductase inhibitors':ab,ti OR 'inhibitors, hydroxymethylglutaryl-coa reductase':ab,ti OR 'reductase inhibitors, hydroxymethylglutaryl-coa':ab,ti OR 'inhibitors, hmg-coa reductase':ab,ti OR 'inhibitors, hmg coa reductase':ab,ti OR 'reductase inhibitors, hmg-coa':ab,ti OR 'hmg-coa reductase inhibitors':ab,ti OR 'hmg coa reductase inhibitors':ab,ti OR 'statins, hmg-coa':ab,ti OR 'hmg-coa statins':ab,ti OR 'statins, hmg coa':ab,ti OR 'inhibitors, hydroxymethylglutaryl-coa':ab,ti OR 'hydroxymethylglutaryl-coa inhibitors':ab,ti OR 'inhibitors, hydroxymethylglutaryl coa':ab,ti OR statins:ab,ti OR 'inhibitors, hydroxymethylglutaryl-coenzyme a':ab,ti OR 'hydroxymethylglutaryl-coenzyme a inhibitors':ab,ti OR 'inhibitors, hydroxymethylglutaryl coenzyme a':ab,ti OR 'hmg-coa reductase inhibitor*':ab,ti OR 'lipid-lowering drug':ab,ti OR 'lipid-lowering agent':ab,ti OR simvastatin:ab,ti OR pravastatin:ab,ti OR atorvastatin:ab,ti OR rosuvastatin:ab,ti OR fluvastatin:ab,ti OR lovastatin:ab,ti OR pitavastatin:ab,ti OR statin:ab,ti OR minocycline:ab,ti OR 'minox 50':ab,ti OR aknemin:ab,ti OR 'aknin mino':ab,ti OR aknosan:ab,ti OR mynocine:ab,ti OR 'apo minocycline':ab,ti OR arestin:ab,ti OR blemix:ab,ti OR cyclomin:ab,ti OR cyclops:ab,ti OR dentomycin:ab,ti OR dynacin:ab,ti OR 'icht oral':ab,ti OR klinomycin:ab,ti OR lederderm:ab,ti OR mestacine:ab,ti OR minakne:ab,ti OR 'mino wolff':ab,ti OR 'mino wolff or minocin':ab,ti OR 'minocin mr':ab,ti OR minoclir:ab,ti OR 'minocycline hydrochloride':ab,ti OR 'hydrochloride, minocycline':ab,ti OR 'minocycline monohydrochloride':ab,ti OR 'monohydrochloride, minocycline':ab,ti OR minolis:ab,ti OR minomycin:ab,ti OR minoplus:ab,ti OR minotab:ab,ti OR akamin:ab,ti OR 'akne puren':ab,ti OR pioglitazone:ab,ti OR 'u 72107a':ab,ti OR 'u72,107a':ab,ti OR 'u-72107a':ab,ti OR 'u72107a':ab,ti OR 'ad 4833':ab,ti OR 'ad-4833':ab,ti OR 'ad4833':ab,ti OR 'pioglitazone hydrochloride':ab,ti OR actos:ab,ti OR 'fatty acids':ab,ti OR 'fatty acids, omega 3':ab,ti OR 'fatty acid':ab,ti OR fats:ab,ti OR omega‐3:ab,ti OR 'omega‐3 fatty acids':ab,ti OR pufa*:ab,ti OR polyunsaturated:ab,ti OR efa:ab,ti OR epa:ab,ti OR 'eicosapentaenoic acid*':ab,ti OR ala:ab,ti OR alas:ab,ti OR 'alpha-linolenic acid*':ab,ti OR 'alphalinolenic acid*':ab,ti OR dha:ab,ti OR dhas:ab,ti OR 'docosahexaenoic acid*':ab,ti OR dpa:ab,ti OR 'docosapentanoic acid*':ab,ti OR 'n‐3‐fatty‐acid*':ab,ti OR 'flaxseed oil':ab,ti OR 'linseed oil':ab,ti OR 'fish oil*':ab,ti OR 'salmon oil':ab,ti OR 'cod liver oil':ab,ti OR 'mackerel oil':ab,ti OR 'tuna* oil':ab,ti OR 'tuna fish oil':ab,ti OR 'blackcurrant oil':ab,ti OR 'canola oil':ab,ti OR 'rapeseed oil':ab,ti OR 'mustard oil*':ab,ti OR 'walnut oil':ab,ti OR 'wheat germ oil':ab,ti OR 'dental oil*':ab,ti OR 'primrose oil':ab,ti OR acetylcysteine:ab,ti OR 'n acetyl l cysteine':ab,ti OR 'n acetylcysteine':ab,ti OR nac:ab,ti  #2 'depressive disorder':ab,ti OR 'depressive disorders':ab,ti OR 'disorder, depressive':ab,ti OR 'disorders, depressive':ab,ti OR 'neurosis, depressive':ab,ti OR 'depressive neuroses':ab,ti OR 'depressive neurosis':ab,ti OR 'neuroses, depressive':ab,ti OR 'depression, endogenous':ab,ti OR 'depressions, endogenous':ab,ti OR 'endogenous depression':ab,ti OR 'endogenous depressions':ab,ti OR 'depressive syndrome':ab,ti OR 'depressive syndromes':ab,ti OR 'syndrome, depressive':ab,ti OR 'syndromes, depressive':ab,ti OR 'depression, neurotic':ab,ti OR 'depressions, neurotic':ab,ti OR 'neurotic depression':ab,ti OR 'neurotic depressions':ab,ti OR melancholia:ab,ti OR melancholies:ab,ti OR 'unipolar depression':ab,ti OR 'depression, unipolar':ab,ti OR 'depressions, unipolar':ab,ti OR 'unipolar depressions':ab,ti OR depression:ab,ti  #3 'randomized controlled trials':ab,ti OR 'randomized controlled trial':ab,ti OR randomized:ab,ti OR randomised:ab,ti OR rct:ab,ti OR random*:ab,ti OR allocate:ab,ti OR assign*:ab,ti OR trial:ab,ti  #4 #1 and #2 and #3 |
| Pubmed | #1 (Anti-inflammatory Agents[MeSH Terms]) OR ("Anti-inflammatory Agents" [Title/Abstract] OR "Anti Inflammatory Agents" [Title/Abstract] OR "Agents, Antiinflammatory" [Title/Abstract] OR Antiinflammatories [Title/Abstract] OR "Antiinflammatory Agents" [Title/Abstract] OR "Agents, Anti-Inflammatory" [Title/Abstract] OR "Agents, Anti Inflammatory" [Title/Abstract] OR Anti-Inflammatories [Title/Abstract] OR "Anti Inflammatories" [Title/Abstract] OR "Anti-inflammatory agents" [Title/Abstract] OR "Anti-inflammatory drugs" [Title/Abstract] OR "Anti-inflammatory drug" [Title/Abstract])  #2 (((Anti-Inflammatory Agents, Non-Steroidal[MeSH Terms]) OR Cyclooxygenase Inhibitors[MeSH Terms]) OR Cyclooxygenase 2 Inhibitors[MeSH Terms]) OR ("Anti-Inflammatory Agents, Non-Steroidal" [Title/Abstract] OR nsaid [Title/Abstract] OR "Antiinflammatory Agents, Non Steroidal" [Title/Abstract] OR NSAIDs [Title/Abstract] OR "Non-Steroidal Anti-Inflammatory Agents" [Title/Abstract] OR "Non Steroidal Anti Inflammatory Agents" [Title/Abstract] OR "Nonsteroidal Anti-Inflammatory Agents" [Title/Abstract] OR "Nonsteroidal Anti Inflammatory Agents" [Title/Abstract] OR "Anti Inflammatory Agents, Nonsteroidal" [Title/Abstract] OR "Antiinflammatory Agents, Nonsteroidal" [Title/Abstract] OR "Nonsteroidal Antiinflammatory Agents" [Title/Abstract] OR "Analgesics, Anti-Inflammatory" [Title/Abstract] OR "Anti-Inflammatory Analgesics" [Title/Abstract] OR "Aspirin-Like Agents" [Title/Abstract] OR "Aspirin Like Agents" [Title/Abstract] OR "Cyclooxygenase Inhibitors" [Title/Abstract] OR "Inhibitors, Cyclo-Oxygenase" [Title/Abstract] OR "Inhibitors, Cyclo Oxygenase" [Title/Abstract] OR "Inhibitors, Cyclooxygenase" [Title/Abstract] OR "Prostaglandin Synthesis Antagonists" [Title/Abstract] OR "Antagonists, Prostaglandin Synthesis" [Title/Abstract] OR "Inhibitors, Prostaglandin-Endoperoxide Synthase" [Title/Abstract] OR "Inhibitors, Prostaglandin Endoperoxide Synthase" [Title/Abstract] OR "Prostaglandin Endoperoxide Synthase Inhibitors" [Title/Abstract] OR "Prostaglandin Synthase Inhibitors" [Title/Abstract] OR "Cyclo-Oxygenase Inhibitors" [Title/Abstract] OR "Cyclo Oxygenase Inhibitors" [Title/Abstract] OR "Inhibitors, Prostaglandin Synthase" [Title/Abstract] OR "Cyclooxygenase 2 Inhibitors" [Title/Abstract] OR "Inhibitors, Cyclooxygenase 2" [Title/Abstract] OR "Cyclooxygenase-2 Inhibitors" [Title/Abstract] OR "Inhibitors, Cyclooxygenase-2" [Title/Abstract] OR Coxibs [Title/Abstract] OR "COX-2 Inhibitors" [Title/Abstract] OR "COX 2 Inhibitors" [Title/Abstract] OR "Inhibitors, COX-2" [Title/Abstract] OR "COX2 Inhibitors" [Title/Abstract] OR "Inhibitors, COX2"[Title/Abstract] OR "non-steroidal anti inflammat*" [Title/Abstract] OR "non-steroidal anti-inflammat*" [Title/Abstract] OR cyclooxygenase [Title/Abstract] OR cyclo-oxygenase [Title/Abstract] OR aspirin [Title/Abstract] OR acetylsalicyl* [Title/Abstract] OR "carbasalate calcium" [Title/Abstract] OR diflunisal [Title/Abstract] OR aceclofenac [Title/Abstract] OR alclofenac [Title/Abstract] OR diclofenac [Title/Abstract] OR indomethacin [Title/Abstract] OR indomethacin [Title/Abstract] OR sulindac [Title/Abstract] OR piroxicam [Title/Abstract] OR dexibuprofen [Title/Abstract] OR dexketoprofen [Title/Abstract] OR fenoprofen [Title/Abstract] OR flurbiprofen [Title/Abstract] OR ibuprofen [Title/Abstract] OR ketoprofen [Title/Abstract] OR naproxen [Title/Abstract] OR tiapro* [Title/Abstract] OR metamizol [Title/Abstract] OR phenylbutazone [Title/Abstract] OR phenazone [Title/Abstract] OR propyphenazone [Title/Abstract] OR etoricoxib [Title/Abstract] OR nabumeton [Title/Abstract] OR parecoxib [Title/Abstract] OR rofecoxib [Title/Abstract] OR celecoxib [Title/Abstract] OR valdecoxib [Title/Abstract] OR lumiracoxib [Title/Abstract] OR vioxx [Title/Abstract] OR celebrex [Title/Abstract] OR bextra [Title/Abstract] OR prexige [Title/Abstract] OR arcoxia [Title/Abstract] OR etodolac [Title/Abstract] OR floctafenine [Title/Abstract] OR meclofenam* [Title/Abstract] OR meloxicam [Title/Abstract] OR oxaprozin [Title/Abstract] OR piroxicam [Title/Abstract] OR tenoxicam [Title/Abstract] OR tolmetin [Title/Abstract] OR acetaminophen [Title/Abstract] OR paracetamol [Title/Abstract] OR rofecoxib [Title/Abstract])  #3 ((Adrenal Cortex Hormone[MeSH Terms]) OR glucocorticoids[MeSH Terms]) OR ("Adrenal Cortex Hormones" [Title/Abstract] OR "Hormones, Adrenal Cortex" [Title/Abstract] OR Corticosteroids [Title/Abstract] OR Corticoids [Title/Abstract] OR glucocorticoids [Title/Abstract] OR Glucocorticoid [Title/Abstract] OR "Glucocorticoid Effect" [Title/Abstract] OR "Effect, Glucocorticoid" [Title/Abstract] OR "Glucorticoid Effects" [Title/Abstract] OR "Effects, Glucorticoid" [Title/Abstract] OR corticoid [Title/Abstract] OR adrenocorticoid [Title/Abstract] OR hydrocortisone [Title/Abstract] OR chronocort [Title/Abstract] OR plenadren [Title/Abstract] OR beclomethasone [Title/Abstract] OR betamethasone [Title/Abstract] OR budesonide [Title/Abstract] OR cortisone [Title/Abstract] OR dexamethasone [Title/Abstract] OR methylprednisolone [Title/Abstract] OR prednisolone [Title/Abstract] OR prednisone [Title/Abstract] OR triamcinolone [Title/Abstract] OR meprednisone [Title/Abstract] OR betametasone [Title/Abstract] OR dexametasone[Title/Abstract])  #4 (Antibodies, Monoclonal[MeSH Terms]) OR ("cytokine inhibitor" [Title/Abstract] OR "TNF inhibitor" [Title/Abstract] OR "tumour necrosis factor inhibitor" [Title/Abstract] OR "tumor necrosis factor inhibitor infliximab" [Title/Abstract] OR etanercept [Title/Abstract] OR adalimumab [Title/Abstract] OR ustekinumab [Title/Abstract] OR "Antibodies, Monoclonal" [Title/Abstract] OR "Monoclonal Antibodies" [Title/Abstract])  #5 (Hydroxymethylglutaryl-CoA Reductase Inhibitors[MeSH Terms]) OR ("Hydroxymethylglutaryl-CoA Reductase Inhibitors" [Title/Abstract] OR "Hydroxymethylglutaryl CoA Reductase Inhibitors" [Title/Abstract] OR "Inhibitors, Hydroxymethylglutaryl-CoA Reductase" [Title/Abstract] OR "Reductase Inhibitors, Hydroxymethylglutaryl-CoA" [Title/Abstract] OR "Inhibitors, HMG-CoA Reductase" [Title/Abstract] OR "Inhibitors, HMG CoA Reductase" [Title/Abstract] OR "Reductase Inhibitors, HMG-CoA" [Title/Abstract] OR "HMG-CoA Reductase Inhibitors" [Title/Abstract] OR "HMG CoA Reductase Inhibitors" [Title/Abstract] OR "Statins, HMG-CoA" [Title/Abstract] OR "HMG-CoA Statins" [Title/Abstract] OR "Statins, HMG CoA" [Title/Abstract] OR "Inhibitors, Hydroxymethylglutaryl-CoA" [Title/Abstract] OR "Hydroxymethylglutaryl-CoA Inhibitors" [Title/Abstract] OR "Inhibitors, Hydroxymethylglutaryl CoA" [Title/Abstract] OR Statins [Title/Abstract] OR "Inhibitors, Hydroxymethylglutaryl-Coenzyme A" [Title/Abstract] OR "Hydroxymethylglutaryl-Coenzyme A Inhibitors" [Title/Abstract] OR "Inhibitors, Hydroxymethylglutaryl Coenzyme A" [Title/Abstract] OR "HMG-CoA reductase inhibitor*" [Title/Abstract] OR "lipid-lowering drug" [Title/Abstract] OR "lipid-lowering agent" [Title/Abstract] OR simvastatin [Title/Abstract] OR pravastatin [Title/Abstract] OR atorvastatin [Title/Abstract] OR rosuvastatin [Title/Abstract] OR Fluvastatin [Title/Abstract] OR Lovastatin [Title/Abstract] OR Pitavastatin [Title/Abstract] OR statin[Title/Abstract])  #6 (minocycline[MeSH Terms]) OR (minocycline [Title/Abstract] OR "Minox 50" [Title/Abstract] OR Aknemin [Title/Abstract] OR Aknin-Mino [Title/Abstract] OR "Aknin Mino" [Title/Abstract] OR Aknosan [Title/Abstract] OR Mynocine [Title/Abstract] OR Apo-Minocycline [Title/Abstract] OR "Apo Minocycline" [Title/Abstract] OR Arestin [Title/Abstract] OR Blemix [Title/Abstract] OR Cyclomin [Title/Abstract] OR Cyclops [Title/Abstract] OR Dentomycin [Title/Abstract] OR Dynacin [Title/Abstract] OR Icht-Oral [Title/Abstract] OR "Icht Oral" [Title/Abstract] OR Klinomycin [Title/Abstract] OR Lederderm [Title/Abstract] OR Mestacine [Title/Abstract] OR Minakne [Title/Abstract] OR Mino-Wolff [Title/Abstract] OR "Mino Wolff" [Title/Abstract] OR Minocin [Title/Abstract] OR "Minocin MR" [Title/Abstract] OR Minoclir [Title/Abstract] OR "Minocycline Hydrochloride" [Title/Abstract] OR "Hydrochloride, Minocycline" [Title/Abstract] OR "Minocycline Monohydrochloride" [Title/Abstract] OR "Monohydrochloride, Minocycline" [Title/Abstract] OR "Minocycline, (4R-(4 alpha,4a beta,5a beta,12a beta))-Isomer" [Title/Abstract] OR Minolis [Title/Abstract] OR Minomycin [Title/Abstract] OR Minoplus [Title/Abstract] OR Minotab [Title/Abstract] OR Akamin [Title/Abstract] OR Akne-Puren [Title/Abstract] OR "Akne Puren" [Title/Abstract])  #7 (pioglitazone [MeSH Terms]) OR (Pioglitazone [Title/Abstract] OR "5-(4-(2-(5-Ethyl-2-pyridyl)ethoxy)benzyl)-2,4-thiazolidinedione" [Title/Abstract] OR "U 72107A" [Title/Abstract] OR "U72,107A" [Title/Abstract] OR U-72107A [Title/Abstract] OR U72107A [Title/Abstract] OR "AD 4833" [Title/Abstract] OR AD-4833 [Title/Abstract] OR AD4833 [Title/Abstract] OR "Pioglitazone Hydrochloride" [Title/Abstract] OR Actos[Title/Abstract])  #8 ((((Fatty Acids, Omega-3[MeSH Terms]) OR fish oils[MeSH Terms]) OR docosahexaenoic acids[MeSH Terms]) OR eicosapentaenoic acid[MeSH Terms]) OR ("fatty acids" [Title/Abstract] OR "fatty acids, omega 3" [Title/Abstract] OR "fatty acid" [Title/Abstract] OR fats [Title/Abstract] OR omega-3 [Title/Abstract] OR "omega-3 fatty acids" [Title/Abstract] OR PUFA* [Title/Abstract] OR Polyunsaturated [Title/Abstract] OR EFA [Title/Abstract] OR EPA [Title/Abstract] OR "eicosapentaenoic acid*" [Title/Abstract] OR ALA [Title/Abstract] OR ALAs [Title/Abstract] OR "alpha-linolenic acid*" [Title/Abstract] OR "alphalinolenic acid*" [Title/Abstract] OR DHA [Title/Abstract] OR DHAs [Title/Abstract] OR "docosahexaenoic acid*" [Title/Abstract] OR DPA [Title/Abstract] OR "docosapentanoic acid*" [Title/Abstract] OR n-3-fatty-acid* [Title/Abstract] OR "flaxseed oil"[Title/Abstract] OR "linseed oil"[Title/Abstract] OR "fish oil*"[Title/Abstract] OR "salmon oil"[Title/Abstract] OR "cod liver oil"[Title/Abstract] OR "mackerel oil"[Title/Abstract] OR "tuna* oil"[Title/Abstract] OR "tuna fish oil"[Title/Abstract] OR "blackcurrant oil"[Title/Abstract] OR "canola oil"[Title/Abstract] OR "rapeseed oil"[Title/Abstract] OR "mustard oil*"[Title/Abstract] OR "walnut oil"[Title/Abstract] OR "wheat germ oil"[Title/Abstract] OR "dental oil*"[Title/Abstract] OR "primrose oil"[Title/Abstract])  #9 (Acetylcysteine [MeSH Terms]) OR (Acetylcysteine [Title/Abstract] OR N-Acetyl-L-cysteine [Title/Abstract] OR "N Acetyl L cysteine" [Title/Abstract] OR N-Acetylcysteine [Title/Abstract] OR "N Acetylcysteine" [Title/Abstract] OR NAC[Title/Abstract])  #10 #1 OR #2 OR #3 OR #4 OR #5 OR #6 OR #7 OR #8 OR #9  #11 (Depressive Disorder[MeSH Terms]) OR ("Depressive Disorder" [Title/Abstract] OR "Depressive Disorders" [Title/Abstract] OR "Disorder, Depressive" [Title/Abstract] OR "Disorders, Depressive" [Title/Abstract] OR "Neurosis, Depressive" [Title/Abstract] OR "Depressive Neuroses" [Title/Abstract] OR "Depressive Neurosis" [Title/Abstract] OR "Neuroses, Depressive" [Title/Abstract] OR "Depression, Endogenous" [Title/Abstract] OR "Depressions, Endogenous" [Title/Abstract] OR "Endogenous Depression" [Title/Abstract] OR "Endogenous Depressions" [Title/Abstract] OR "Depressive Syndrome" [Title/Abstract] OR "Depressive Syndromes" [Title/Abstract] OR "Syndrome, Depressive" [Title/Abstract] OR "Syndromes, Depressive" [Title/Abstract] OR "Depression, Neurotic" [Title/Abstract] OR "Depressions, Neurotic" [Title/Abstract] OR "Neurotic Depression" [Title/Abstract] OR "Neurotic Depressions" [Title/Abstract] OR Melancholia [Title/Abstract] OR Melancholies [Title/Abstract] OR "Unipolar Depression" [Title/Abstract] OR "Depression, Unipolar" [Title/Abstract] OR "Depressions, Unipolar" [Title/Abstract] OR "Unipolar Depressions" [Title/Abstract] OR Depression[Title/Abstract])  #12 ("Randomized Controlled Trials" [Title/Abstract] OR "Randomized Controlled Trial" [Title/Abstract] OR Randomized [Title/Abstract] OR randomised [Title/Abstract] OR RCT [Title/Abstract] OR random* [Title/Abstract] OR allocated [Title/Abstract] OR assign* [Title/Abstract] OR trial [Title/Abstract])  #13 #10 AND #11 AND #12 |
| Web of Science | #1 TS=("Anti‐Inflammatory Agents" OR "Anti Inflammatory Agents" OR "Agents, Antiinflammatory" OR Antiinflammatories OR "Antiinflammatory Agents" OR "Agents, Anti-Inflammatory" OR "Agents, Anti Inflammatory" OR Anti-Inflammatories OR "Anti Inflammatories" OR "Anti-inflammatory agents" OR "Anti-inflammatory drugs" OR "Anti-inflammatory drug")  #2 TS=("Anti-Inflammatory Agents, Non-Steroidal" OR nsaid OR "Antiinflammatory Agents, Non Steroidal" OR NSAIDs OR "Non-Steroidal Anti-Inflammatory Agents" OR "Non Steroidal Anti Inflammatory Agents" OR "Nonsteroidal Anti-Inflammatory Agents" OR "Nonsteroidal Anti Inflammatory Agents" OR "Anti Inflammatory Agents, Nonsteroidal" OR "Antiinflammatory Agents, Nonsteroidal" OR "Nonsteroidal Antiinflammatory Agents" OR "Analgesics, Anti-Inflammatory" OR "Anti-Inflammatory Analgesics" OR "Aspirin-Like Agents" OR "Aspirin Like Agents" OR "Cyclooxygenase Inhibitors" OR "Inhibitors, Cyclo-Oxygenase" OR "Inhibitors, Cyclo Oxygenase" OR "Inhibitors, Cyclooxygenase" OR "Prostaglandin Synthesis Antagonists" OR "Antagonists, Prostaglandin Synthesis" OR "Inhibitors, Prostaglandin-Endoperoxide Synthase" OR "Inhibitors, Prostaglandin Endoperoxide Synthase" OR "Prostaglandin Endoperoxide Synthase Inhibitors" OR "Prostaglandin Synthase Inhibitors" OR "Cyclo-Oxygenase Inhibitors" OR "Cyclo Oxygenase Inhibitors" OR "Inhibitors, Prostaglandin Synthase" OR "Cyclooxygenase 2 Inhibitors" OR "Inhibitors, Cyclooxygenase 2" OR "Cyclooxygenase-2 Inhibitors" OR "Inhibitors, Cyclooxygenase-2" OR Coxibs OR "COX-2 Inhibitors" OR "COX 2 Inhibitors" OR "Inhibitors, COX-2" OR "COX2 Inhibitors" OR "Inhibitors, COX2" OR "non‐steroidal anti inflammat*" OR "non‐steroidal anti‐inflammat*" OR cyclooxygenase OR cyclo‐oxygenase OR aspirin OR acetylsalicyl* OR "carbasalate calcium" OR diflunisal OR aceclofenac OR alclofenac OR diclofenac OR ndometacin OR indomethacin OR sulindac OR piroxicam OR dexibuprofen OR dexketoprofen OR fenoprofen OR flurbiprofen OR ibuprofen OR ketoprofen OR naproxen OR tiapro* OR metamizol OR phenylbutazone OR phenazone OR propyphenazone OR etoricoxib OR nabumeton OR parecoxib OR rofecoxib OR celecoxib OR valdecoxib OR lumiracoxib OR vioxx OR celebrex OR bextra OR prexige OR arcoxia OR etodolac OR floctafenine OR meclofenam* OR meloxicam OR oxaprozin OR piroxicam OR tenoxicam OR tolmetin OR acetaminophen OR paracetamol OR rofecoxib)  #3 TS=("Adrenal Cortex Hormones" OR "Hormones, Adrenal Cortex" OR Corticosteroids OR Corticoids OR glucocorticoids OR Glucocorticoid OR "Glucocorticoid Effect" OR "Effect, Glucocorticoid" OR "Glucorticoid Effects" OR "Effects, Glucorticoid" OR corticoid OR adrenocorticoid OR hydrocortisone OR chronocort OR plenadren OR beclomethasone OR betamethasone OR budesonide OR cortisone OR dexamethasone OR methylprednisolone OR prednisolone OR prednisone OR triamcinolone OR meprednisone OR betametasone OR dexametasone)  #4 TS=("cytokine inhibitor" OR "TNF inhibitor" OR "tumour necrosis factor inhibitor" OR "tumor necrosis factor inhibitor infliximab" OR etanercept OR adalimumab OR ustekinumab OR "Antibodies, Monoclonal" OR "Monoclonal Antibodies")  #5 TS=("Hydroxymethylglutaryl-CoA Reductase Inhibitors" OR "Hydroxymethylglutaryl CoA Reductase Inhibitors" OR "Inhibitors, Hydroxymethylglutaryl-CoA Reductase" OR "Reductase Inhibitors, Hydroxymethylglutaryl-CoA" OR "Inhibitors, HMG-CoA Reductase" OR "Inhibitors, HMG CoA Reductase" OR "Reductase Inhibitors, HMG-CoA" OR "HMG-CoA Reductase Inhibitors" OR "HMG CoA Reductase Inhibitors" OR "Statins, HMG-CoA" OR "HMG-CoA Statins" OR "Statins, HMG CoA" OR "Inhibitors, Hydroxymethylglutaryl-CoA" OR "Hydroxymethylglutaryl-CoA Inhibitors" OR "Inhibitors, Hydroxymethylglutaryl CoA" OR Statins OR "Inhibitors, Hydroxymethylglutaryl-Coenzyme A" OR "Hydroxymethylglutaryl-Coenzyme A Inhibitors" OR "Inhibitors, Hydroxymethylglutaryl Coenzyme A" OR "HMG-CoA reductase inhibitor*" OR "lipid-lowering drug" OR "lipid-lowering agent" OR simvastatin OR pravastatin OR atorvastatin OR rosuvastatin OR Fluvastatin OR Lovastatin OR Pitavastatin OR statin)  #6 TS=(minocycline OR "Minox 50" OR Aknemin OR Aknin-Mino OR "Aknin Mino" OR Aknosan OR Mynocine OR Apo-Minocycline OR "Apo Minocycline" OR Arestin OR Blemix OR Cyclomin OR Cyclops OR Dentomycin OR Dynacin OR Icht-Oral OR "Icht Oral" OR Klinomycin OR Lederderm OR Mestacine OR Minakne OR Mino-Wolff OR "Mino Wolff" OR Minocin OR "Minocin MR" OR Minoclir OR "Minocycline Hydrochloride" OR "Hydrochloride, Minocycline" OR "Minocycline Monohydrochloride" OR "Monohydrochloride, Minocycline" OR Minolis OR Minomycin OR Minoplus OR Minotab OR Akamin OR Akne-Puren OR "Akne Puren")  #7 TS=(Pioglitazone OR "U 72107A" OR "U72,107A" OR "U-72107A" OR "U72107A" OR "AD 4833" OR "AD-4833" OR "AD4833" OR "Pioglitazone Hydrochloride" OR Actos)  #8 TS=("fatty acids" OR "fatty acids, omega 3" OR "fatty acid" OR fats OR omega‐3 OR "omega‐3 fatty acids" OR PUFA* OR Polyunsaturated OR EFA OR EPA OR "eicosapentaenoic acid*" OR ALA OR ALAs OR "alpha-linolenic acid*" OR "alphalinolenic acid*" OR DHA OR DHAs OR "docosahexaenoic acid*" OR DPA OR "docosapentanoic acid*" OR "n‐3‐fatty‐acid*" OR "flaxseed oil" OR "linseed oil" OR "fish oil*" OR "salmon oil" OR "cod liver oil" OR "mackerel oil" OR "tuna* oil" OR "tuna fish oil" OR "blackcurrant oil" OR "canola oil" OR "rapeseed oil" OR "mustard oil*" OR "walnut oil" OR "wheat germ oil" OR "dental oil*" OR "primrose oil")  #9 TS=(Acetylcysteine OR N-Acetyl-L-cysteine OR "N Acetyl L cysteine" OR N-Acetylcysteine OR "N Acetylcysteine" OR NAC)  # 10 #9 OR #8 OR #7 OR #6 OR #5 OR #4 OR #3 OR #2 OR #1  #11 TS=("Depressive Disorder" OR "Depressive Disorders" OR "Disorder, Depressive" OR "Disorders, Depressive" OR "Neurosis, Depressive" OR "Depressive Neuroses" OR "Depressive Neurosis" OR "Neuroses, Depressive" OR "Depression, Endogenous" OR "Depressions, Endogenous" OR "Endogenous Depression" OR "Endogenous Depressions" OR "Depressive Syndrome" OR "Depressive Syndromes" OR "Syndrome, Depressive" OR "Syndromes, Depressive" OR "Depression, Neurotic" OR "Depressions, Neurotic" OR "Neurotic Depression" OR "Neurotic Depressions" OR Melancholia OR Melancholies OR "Unipolar Depression" OR "Depression, Unipolar" OR "Depressions, Unipolar" OR "Unipolar Depressions" OR Depression)  # 12 TS=("Randomized Controlled Trials" OR "Randomized Controlled Trial" OR Randomized OR randomised OR RCT OR random* OR allocated OR assign* OR trial)  # 13 #12 AND #11 AND #10 |
