# Supplementary material for: Efficacy and acceptability of anti-inflammatory agents in major depressive disorder: a systematic review and meta-analysis
Source: Front Psychiatry. 2024 May 28;15:1407529. doi: 10.3389/fpsyt.2024.1407529 (PMC11165078; doi:10.3389/fpsyt.2024.1407529)
Supplement: Supplementary file 1 [file DataSheet_1.zip › Supplementary Table 2.DOCX]

| Patient | Individuals aged 18 years and older with MDD of both genders |
| --- | --- |
| Intervention | Anti-inflammatory drugs alone (anti-inflammatory agent vs. placebo) or in combination with antidepressant drugs (anti-inflammatory agent + antidepressant drug vs. placebo + antidepressant drug) |
| Comparison | Placebo alone or placebo in combination with anti-depressant drugs |
| Outcome | Our primary outcomes comprise efficacy and acceptability: Efficacy is measured by the response rate, indicating patients with a ≥50% reduction in the total score of standardized depression assessment scales. Acceptability is measured by the rate of patient dropouts due to all-causes, reflecting both efficacy and tolerability;  Secondary outcomes include remission rate, defined as MADRS≤7, HAMD≤7, GDS≤11, or BDI-II≤8 at the end of the trial, and the proportion of patients who dropped out early due to adverse events (AE) |
