# Supplementary material for: Efficacy and acceptability of anti-inflammatory agents in major depressive disorder: a systematic review and meta-analysis
Source: Front Psychiatry. 2024 May 28;15:1407529. doi: 10.3389/fpsyt.2024.1407529 (PMC11165078; doi:10.3389/fpsyt.2024.1407529)
Supplement: Supplementary file 1 [file DataSheet_1.zip › Supplementary Table 4.DOCX]

| ***** Denotes that data are missing from the published or unpublished data and not reportable here. Empty cells have not been extracted. | | | | | | | | | ***Efficacy*** | | | | | ***Acceptability*** | |
| --- | --- | --- | --- | --- | --- | --- | --- | --- | --- | --- | --- | --- | --- | --- | --- |
| **StudyID** | **Year_ Published** | **Drug** | **Treatment** | **Severity** | **Age(mean±SD)** | **Sex (male/ female)** | **No_ randomized** | **Weeks** | **Follow-up** | **Responders** | **Remitters** | **Definition of response** | **Definition of remission** | **Dropouts_total** | **Dropouts_AE** |
| **Omega-3** | | | | | | | | | | | | | | | |
| Mischoulon 2022 | 2022 | Omega-3 | EPA 1-4g/d | IDS-C30≥25 | 41.60±14.60/44.50±15.20/45.80±13.00 | 12/34 | 46 | 12 | 35 | 16 | * | 50% IDS-C30 decrease | * | 11 | * |
| Mischoulon 2022 | 2022 | Placebo | Placebo | IDS-C30≥25 | 50.30±12.00 | 3/12 | 15 | 12 | 10 | 4 | * | 50% IDS-C30 decrease | * | 5 | * |
| Park 2015 | 2015 | Omega-3 | EPA 1.14g/d + DHA 0.6g/d + TAU | CES-D-K≥25 | 43.50±3.72 | 4/14 | 18 | 12 | 12 | * | * | * | * | 6 | * |
| Park 2015 | 2015 | Placebo | Placebo + TAU | CES-D-K≥25 | 39.41±3.58 | 4/13 | 17 | 12 | 12 | * | * | * | * | 5 | * |
| Yang 2022 | 2022 | Omega-3 | EPA 1440mg/d + DHA 960mg/d + Venlafaxin 75-225mg/d | HAMD-24≥20 | 26.33±8.07 | 15/21 | 36 | 12 | 21 | 13 | 3 | 50%HAMD decrease | HAMD ≤7 at end of treatment | 15 | * |
| Yang 2022 | 2022 | Placebo | Placebo + Venlafaxin 75-225mg/d | HAMD-24≥20 | 27.11±8.14 | 11/25 | 36 | 12 | 27 | 12 | 4 | 50%HAMD decrease | HAMD ≤7 at end of treatment | 9 | * |
| Su 2003 | 2003 | Omega-3 | o-3 PUFAs 9.6g/d + TAU | HAMD-21≥18 | 35.20±11.60 | 4/10 | 14 | 8 | 12 | * | * | * | * | 2 | * |
| Su 2003 | 2003 | Placebo | Placebo + TAU | HAMD-21≥18 | 42.30±10.70 | 6/8 | 14 | 8 | 10 | * | * | * | * | 4 | * |
| Rondanelli 2010 | 2010 | Omega-3 | n-3 PUFA 2.5g/d | GDS>10 | 84.90±6.90 | 0/22 | 22 | 8 | 22 | * | 9 | * | GDS <11 at end of treatment | 0 | * |
| Rondanelli 2010 | 2010 | Placebo | Placebo | GDS>10 | 83.00±7.30 | 0/24 | 24 | 8 | 24 | * | 4 | * | GDS <11 at end of treatment | 0 | * |
| Nemets 2002 | 2002 | Omega-3 | E-EPA 2g/d + TAU | HAMD≥18 | 54.20±13.90 | 1/9 | 10 | 4 | 10 | 6 | 3 | 50%HAMD decrease | HAMD ≤7 at end of treatment | 0 | * |
| Nemets 2002 | 2002 | Placebo | Placebo + TAU | HAMD≥18 | 52.10±10.20 | 2/8 | 10 | 4 | 9 | 1 | 1 | 50%HAMD decrease | HAMD ≤7 at end of treatment | 1 | * |
| Marangell 2003 | 2003 | Omega-3 | DHA 2g/d | MADRS≥12 | 46.80±11.60 | 4/14 | 18 | 6 | 18 | 5 | * | 50%MADRS decrease | * | * | * |
| Marangell 2003 | 2003 | Placebo | Placebo | MADRS≥12 | 47.90±11.20 | 3/14 | 17 | 6 | 17 | 4 | * | 50%MADRS decrease | * | * | * |
| Grenyer2007 | 2007 | Omega-3 | n-3 PUFA 3g + TAU | HAMD>16 | * | * | 40 | 16 | 32 | * | * | * | * | 8 | * |
| Grenyer2007 | 2007 | Placebo | Placebo + TAU | HAMD>16 | * | * | 43 | 16 | 28 | * | * | * | * | 15 | * |
| Jazayeri2008a | 2008 | Omega-3 | EPA 1g/d + Fluoxetine 20mg/d | HAMD-24>16 | 34.50±11.30 | 7/9 | 20 | 8 | 16 | 13 | * | 50%HAMD decrease | * | 4 | 0 |
| Jazayeri2008a | 2008 | Placebo | Placebo + Fluoxetine 20mg/d | HAMD-24>16 | 35.10±9.40 | 4/12 | 20 | 8 | 16 | 8 | * | 50%HAMD decrease | * | 4 | 1 |
| Jazayeri2008b | 2008 | Omega-3 | EPA 1g/d + Placebo | HAMD-24>16 | 34.50±11.30 | 4/12 | 20 | 8 | 16 | 9 | * | 50%HAMD decrease | * | 4 | 0 |
| Jazayeri2008b | 2008 | Placebo | Placebo + Fluoxetine 20mg/d | HAMD-24>16 | 35.10±9.40 | 4/12 | 20 | 8 | 16 | 8 | * | 50%HAMD decrease | * | 4 | 1 |
| Mischoulon 2009 | 2009 | Omega-3 | EPA 1g/d | HAMD-17≥18 | 43±13 | 1/16 | 17 | 8 | 11 | 5 | 4 | 50%HAMD decrease | HAMD ≤7 at end of treatment | 6 | * |
| Mischoulon 2009 | 2009 | Placebo | Placebo | HAMD-17≥18 | 43±13 | 5/19 | 24 | 8 | 13 | 3 | 2 | 50%HAMD decrease | HAMD ≤7 at end of treatment | 11 | * |
| Lespérance 2011 | 2011 | Omega-3 | EPA 1.05g/d + DHA 0.15g/d | IDS-SR30≥25 | 46.60±11.54 | 75/143 | 218 | 8 | 188 | * | * | * | * | 30 | 7 |
| Lespérance 2011 | 2011 | Placebo | Placebo | IDS-SR30≥25 | 45.40±13.27 | 61/153 | 214 | 8 | 187 | * | * | * | * | 27 | 4 |
| Gertsik 2012 | 2012 | Omega-3 | n-3 PUFA 1.2g/d + Citalopram 20-40mg/d | HAMD-21≥17 | * | * | 18 | 8 | 17 | 8 | 3 | 50%HAMD decrease | HAMD ≤7 at end of treatment | 1 | * |
| Gertsik 2012 | 2012 | Placebo | Placebo + Citalopram 20-40mg/d | HAMD-21≥17 | * | * | 22 | 8 | 15 | 4 | 3 | 50%HAMD decrease | HAMD ≤7 at end of treatment | 7 | * |
| Mischoulon 2015 | 2015 | Omega-3 | EPA 1g/d/DHA 1g/d + TAU | HAMD-17≥15 | 46.20±11.80/46.30±13.70 | 61/70 | 131 | 8 | 118 | 52 | 36 | 50%HAMD decrease | HAMD ≤7 at end of treatment | 13 | * |
| Mischoulon 2015 | 2015 | Placebo | Placebo + TAU | HAMD-17≥15 | 45.00±12.1 | 30/35 | 65 | 8 | 59 | 28 | 19 | 50%HAMD decrease | HAMD ≤7 at end of treatment | 6 | * |
| Silvers 2005 | 2005 | Omega-3 | o-3 PUFAs 3g/d | * | 39.8±11.9 | 18/22 | 40 | 12 | 24 | * | * | * | * | 16 | 3 |
| Silvers 2005 | 2005 | Placebo | Placebo | * | 37.7±13.6 | 18/37 | 37 | 12 | 21 | * | * | * | * | 16 | 1 |
| Moghaddam 2021a | 2021 | Omega-3 | EPA 0.36g/d + DHA 0.24g/d + Sertraline 50mg/d | HAMD≥22 | 33.11±9.65 | 8/11 | 25 | 6 | 19 | * | * | * | * | 6 | * |
| Moghaddam 2021a | 2021 | Placebo | Placebo + Sertraline 50mg/d | HAMD≥22 | 34.59±12.63 | 10/22 | 25 | 6 | 22 | * | * | * | * | 3 | * |
| Moghaddam 2021b | 2021 | Omega-3 | EPA 0.36g/d + DHA 0.24g/d + Venlafaxine 75mg/d | HAMD≥22 | 33.88±11.03 | 7/10 | 25 | 6 | 17 | * | * | * | * | 8 | * |
| Moghaddam 2021b | 2021 | Placebo | Placebo + Venlafaxine 75mg/d | HAMD≥22 | 34.20±11.53 | 9/16 | 25 | 6 | 25 | * | * | * | * | 0 | * |
| Mozaffari-Khosravi 2013 | 2013 | Omega-3 | EPA 1g/d/DHA 1g/d + TAU | HAMD-17≥8 | * | * | 54 | 12 | 21/20 | 6 | 5 | 50%HAMD decrease | HAMD ≤7 at end of treatment | 13 | 5 |
| Mozaffari-Khosravi 2013 | 2013 | Placebo | Placebo + TAU | HAMD-17≥8 | * | * | 27 | 12 | 21 | 0 | 0 | 50%HAMD decrease | HAMD ≤7 at end of treatment | 6 | 4 |
| Peet 2002 | 2002 | Omega-3 | EPA 1-4g/d + TAU | HAMD-17≥15 | 48 | 8/44 | 52 | 12 | 13/16/13 | 17 | * | 50%HAMD decrease | * | 10 | 1 |
| Peet 2002 | 2002 | Placebo | Placebo + TAU | HAMD-17≥15 | 44 | 3/15 | 18 | 12 | 12 | 5 | * | 50%HAMD decrease | * | 6 | 1 |
| Jahangard 2018 | 2018 | Omega-3 | n-3 PUFA 1g/d + Sertraline 50-200mg/d | * | 41.28±11.56 | 17/8 | 25 | 12 | 25 | * | * | * | * | 0 | * |
| Jahangard 2018 | 2018 | Placebo | Placebo + Sertraline 50-200mg/d | * | 43.64±11.29 | 17/8 | 25 | 12 | 25 | * | * | * | * | 0 | * |
| Keshavarz 2018 | 2018 | Omega-3 | EPA 1.08g/d + DHA 0.15g/d | * | 41.00±9.90 | * | 32 | 12 | 24 | * | * | * | * | 8 | * |
| Keshavarz 2018 | 2018 | Placebo | Placebo | * | 44.00±9.50 | * | 33 | 12 | 21 | * | * | * | * | 12 | * |
| **NSAIDs** | | | | | | | | | | | | | | | |
| Müller 2006 | 2006 | NSAIDs | Celecoxib200-400mg/d + Reboxetine 4-10mg/d | * | 44.50±11.60 | 12/8 | 20 | 6 | 10 | * | 9 | * | HAMD ≤7 at end of treatment | 10 | 4 |
| Müller 2006 | 2006 | Placebo | Placebo + Reboxetine 4-10mg/d | * | 44.30±13.50 | 8/12 | 20 | 6 | 8 | * | 4 | * | HAMD ≤7 at end of treatment | 12 | 3 |
| Sepehrmanesh 2017 | 2017 | NSAIDs | Aspirin 16mg/d + Sertraline50-200mg/d | * | 48.90±7.50 | 21/29 | 50 | 8 | 50 | * | * | * | * | 0 | * |
| Sepehrmanesh 2017 | 2017 | Placebo | Placebo + Sertraline 50-200mg/d | * | 47.80±7.30 | 18/32 | 50 | 8 | 50 | * | * | * | * | 0 | * |
| Abbasi 2012 | 2012 | NSAIDs | Celecoxib 400mg/d + Sertraline 200mg/d | HAMD-17≥18 | 35.10±8.00 | 13/7 | 20 | 6 | 19 | 19 | 7 | 50%HAMD decrease | HAMD ≤7 at end of treatment | 1 | * |
| Abbasi 2012 | 2012 | Placebo | Placebo + Sertraline 200mg/d | HAMD-17≥18 | 34.20±6.90 | 14/6 | 20 | 6 | 18 | 10 | 1 | 50%HAMD decrease | HAMD ≤7 at end of treatment | 2 | * |
| Akhondzadeh 2009 | 2009 | NSAIDs | Celecoxib 400mg/d +Fluoxetine 20-40mg/d | HAMD-17≥18 | 34.65±6.76 | 7/13 | 20 | 6 | 19 | 18 | 7 | 50%HAMD decrease | HAMD ≤7 at end of treatment | 1 | * |
| Akhondzadeh 2009 | 2009 | Placebo | Placebo + Fluoxetine 20-40mg/d | HAMD-17≥18 | 34.20±4.96 | 8/12 | 20 | 6 | 18 | 10 | 1 | 50%HAMD decrease | HAMD ≤7 at end of treatment | 2 | * |
| Majd 2015 | 2015 | NSAIDs | Celecoxib 200mg/d + Sertraline 200mg/d | HAMD-17≥18 | 34.70±7.30 | 0/15 | 15 | 8 | 14 | 14 | 8 | 50%HAMD decrease | HAMD ≤7 at end of treatment | 1 | 0 |
| Majd 2015 | 2015 | Placebo | Placebo + Sertraline 200mg/d | HAMD-17≥18 | 36.20±12.7 | 0/15 | 15 | 8 | 9 | 7 | 1 | 50%HAMD decrease | HAMD ≤7 at end of treatment | 6 | 3 |
| Krause 2017 | 2017 | NSAIDs | Celecoxib 400mg/d + Reboxetine 4-10mg/d | HAMD-17≥15 | * | 12/8 | 20 | 6 | 18 | * | 6 | * | HAMD ≤7 at end of treatment | 2 | * |
| Krause 2017 | 2017 | Placebo | Placebo + Reboxetine 4-10mg/d | HAMD-17≥15 | * | 8/12 | 20 | 6 | 14 | * | 3 | * | HAMD ≤7 at end of treatment | 6 | * |
| Baune 2021 | 2021 | NSAIDs | Celecoxib 400mg/d + Vortioxetine | MADRS≥20 | 45.0±2.88 | 27/32 | 59 | 6 | 52 | 18 | 6 | 50%MARDS decrease | MARDS <7 at end of treatment | 7 | 4 |
| Baune 2021 | 2021 | Placebo | Placebo + Vortioxetine | MADRS≥20 | 48.0±4.38 | 23/60 | 60 | 6 | 51 | 17 | 7 | 50%MARDS decrease | MARDS <7 at end of treatment | 9 | 6 |
| Al-Hakeim 2018 | 2018 | NSAIDs | ketoprofen 100mg/d + Sertraline 50mg/d | * | * | * | 28 | 8 | 28 | * | * | * | * | * | * |
| Al-Hakeim 2018 | 2018 | Placebo | Placebo + Sertraline 50mg/d | * | * | * | 16 | 8 | 16 | * | * | * | * | * | * |
| Simon 2021 | 2021 | NSAIDs | Celecoxib 400mg/d + Sertraline 50-100mg/d | MADRS≥20 | * | * | 29 | 6 | 20 | 11 | 5 | 50%MARDS decrease | MARDS ≤7 at end of treatment | 9 | 1 |
| Simon 2021 | 2021 | Placebo | Placebo + Sertraline 50-100mg/d | MADRS≥20 | * | * | 24 | 6 | 23 | 16 | 8 | 50%MARDS decrease | MARDS ≤7 at end of treatment | 1 | 0 |
| **Pioglitazone** | | | | | | | | | | | | | | | |
| Rasgon 2016 | 2016 | Pioglitazone | Pioglitazone 30mg/d + TAU | * | 49.42 | 5/17 | 22 | 12 | 19 | * | * | * | * | 3 | 1 |
| Rasgon 2016 | 2016 | Placebo | Placebo + TAU | * | 43.28 | 6/16 | 20 | 12 | 18 | * | * | * | * | 2 | 0 |
| Robakis2019 | 2019 | Pioglitazone | Pioglitazone 30mg/d + TAU | HAMD-21>7 | 50.24±14.82 | * | 24 | 12 | 19 | * | * | * | * | 3 | * |
| Robakis2019 | 2019 | Placebo | Placebo + TAU | HAMD-21>7 | 44.29±11.45 | * | 19 | 12 | 15 | * | * | * | * | 2 | * |
| Sepanjnia 2012 | 2012 | Pioglitazone | Pioglitazone 30mg/d + Citalopram 20-30mg/d | HAMD-17≥22 | 31.40±5.40 | 6/14 | 20 | 6 | 20 | 19 | 9 | 50%HAMD decrease | HAMD ≤7 at end of treatment | 0 | * |
| Sepanjnia 2012 | 2012 | Placebo | Placebo + Citalopram 20-30mg/d | HAMD-17≥22 | 32.70±5.40 | 5/15 | 20 | 6 | 20 | 8 | 3 | 50%HAMD decrease | HAMD ≤7 at end of treatment | 0 | * |
| **Minocycline** | | | | | | | | | | | | | | | |
| Dean 2017 | 2017 | Minocycline | Minocycline 200mg/d + TAU | MADRS≥25 | 51.0±14.60 | 12/24 | 36 | 12 | 28 | * | * | * | * | 9 | 8 |
| Dean 2017 | 2017 | Placebo | Placebo + TAU | MADRS≥25 | 47.80±14.80 | 12/23 | 35 | 12 | 30 | * | * | * | * | 5 | 1 |
| Husain 2017 | 2017 | Minocycline | Minocycline 200mg/d + TAU | * | * | 11/10 | 21 | 12 | 16 | 10 | * | 50%HAMD decrease | * | 5 | * |
| Husain 2017 | 2017 | Placebo | Placebo + TAU | * | * | 9/11 | 20 | 12 | 18 | 4 | * | 50%HAMD decrease | * | 2 | * |
| Nettis 2021 | 2021 | Minocycline | Minocycline 200mg/d + TAU | HAMD≥14 | * | * | 22 | 4 | 18 | 3 | * | 50%HAMD decrease | * | 4 | 2 |
| Nettis 2021 | 2021 | Placebo | Placebo + TAU | HAMD≥14 | * | * | 22 | 4 | 21 | 2 | * | 50%HAMD decrease | * | 1 | 0 |
| Attwells2021 | 2021 | Minocycline | Minocycline 100mg/d + TAU | HAMD-17≥19 | 36.5±13.4 | 4/8 | 12 | 8 | 12 | 2 | 1 | 50%HAMD decrease | HAMD ≤7 at end of treatment | * | * |
| Attwells2021 | 2021 | Placebo | Placebo + TAU | HAMD-17≥19 | 36.9±12.0 | 2/7 | 9 | 8 | 9 | 3 | 2 | 50%HAMD decrease | HAMD ≤7 at end of treatment | * | * |
| Hellmann-Regen 2022 | 2022 | Minocycline | Minocycline 200mg/d + TAU | HAMD-17≥16 | 44.8±13.6 | 50/31 | 81 | 6 | 69 | 14 | * | 50%MADRS decrease | * | 12 | 2 |
| Hellmann-Regen 2022 | 2022 | Placebo | Placebo + TAU | HAMD-17≥16 | 47.3±12.5 | 39/28 | 87 | 6 | 75 | 21 | * | 50%MADRS decrease | * | 12 | 2 |
| **NACs** | | | | | | | | | | | | | | | |
| Hasebe 2017 | 2017 | NACs | NAC 2g/d + TAU | MADRS≥18 | 50.40±12.50 | 19/39 | 58 | 12 | 47 | * | * | * | * | 11 | * |
| Hasebe 2017 | 2017 | Placebo | Placebo + TAU | MADRS≥18 | 48.80±12.50 | 22/41 | 63 | 12 | 49 | * | * | * | * | 14 | * |
| Berk 2014 | 2014 | NACs | NAC 2g/d + TAU | MADRS≥18 | 49.90±13.00 | 51/84 | 135 | 16 | 98 | 42 | 19 | 50%MARDS decrease | MARDS ≤7 at end of treatment | 29 | 1 |
| Berk 2014 | 2014 | Placebo | Placebo + TAU | MADRS≥18 | 50.50±12.50 | 59/75 | 134 | 16 | 87 | 24 | 6 | 50%MARDS decrease | MARDS ≤7 at end of treatment | 38 | 2 |
| **Corticosteroids** | | | | | | | | | | | | | | | |
| Arana 1995 | 1995 | Corticosteroids | Dexamethasone 4mg/d | HAMD>20 | * | * | 19 | 4days | 19 | 7 | * | 50%HAMD decrease | * | 0 | * |
| Arana 1995 | 1995 | Placebo | Placebo | HAMD>20 | * | * | 18 | 4days | 18 | 1 | * | 50%HAMD decrease | * | 0 | * |
| DeBattista 2000 | 2000 | Corticosteroids | Ovine CRH 1ug/kg/Hydrocortisone 15mg | HAMD≥21 | 46.70±18.00/35.00±10.50 | 6/6 | 12 | 2days | 12 | * | * | * | * | 0 | * |
| DeBattista 2000 | 2000 | Placebo | Placebo | HAMD≥21 | 39.80±10.10 | 3/7 | 10 | 2days | 10 | * | * | * | * | 0 | * |
| Bremner2004 | 2004 | Corticosteroids | dexamethasone 1-2mg/d | * | 44±14 | * | 16 | 2days | 16 | * | * | * | * | * | * |
| Bremner2004 | 2004 | Placebo | Placebo | * | 42±12 | * | 12 | 2days | 12 | * | * | * | * | * | * |
| Otte 2010 | 2010 | Corticosteroids | Fludrocortisone 0.2 mg/d + Escitalopram 10mg/d | HAMD-17≥18 | 36.5±12.7 | 9/15 | 24 | 3 | 22 | * | * | * | * | 2 | 1 |
| Otte 2010 | 2010 | Placebo | Placebo + Escitalopram 10mg/d | HAMD-17≥18 | 34.5±12.7 | 5/8 | 13 | 3 | 12 | * | * | * | * | 1 | 0 |
| **Statins** | | | | | | | | | | | | | | | |
| Ghanizadeh 2013 | 2013 | Statins | Lovastatin 30mg/d + Fluoxetine 40mg/d | HAMD-17≥18 | 32.50±10.20 | 12/22 | 34 | 6 | 30 | * | * | * | * | 4 | * |
| Ghanizadeh 2013 | 2013 | Placebo | Placebo + Fluoxetine 40mg/d | HAMD-17≥18 | 31.70±9.30 | 13/21 | 34 | 6 | 31 | * | * | * | * | 3 | * |
| Haghighi2014 | 2014 | Statins | atorvastatin 20mg/d + Citalopram 40mg/d | HAMD≥25 | 33.07±8.85 | 16/14 | 30 | 12 | 30 | 1 | 0 | 50%HAMD decrease | HAMD ≤7 at end of treatment | 0 | * |
| Haghighi2014 | 2014 | Placebo | Placebo + Citalopram 40mg/d | HAMD≥25 | 31.43±7.96 | 16/14 | 30 | 12 | 30 | 0 | 0 | 50%HAMD decrease | HAMD ≤7 at end of treatment | 0 | * |
| Massardo 2022 | 2022 | Statins | Rosuvastatin 10mg/d + Sertraline | HAMD-17≥15 | 31.5±2.44 | 3/7 | 10 | 12 | 10 | 8 | * | 50%HAMD decrease | * | * | * |
| Massardo 2022 | 2022 | Placebo | Placebo + Sertraline | HAMD-17≥15 | 36.5±2.06 | 3/7 | 10 | 12 | 10 | 6 | * | 50%HAMD decrease | * | * | * |
| Gougol 2015 | 2015 | Statins | Simvastatin 20mg/d + Fluoxetine 20mg/d | HAMD≥22 | 36.40±8.10 | 11/13 | 24 | 6 | 22 | 20 | 13 | 50%HAMD decrease | HAMD ≤7 at end of treatment | 2 | * |
| Gougol 2015 | 2015 | Placebo | Placebo + Fluoxetine 20mg/d | HAMD≥22 | 34.20±10.80 | 8/16 | 24 | 6 | 22 | 13 | 10 | 50%HAMD decrease | HAMD ≤7 at end of treatment | 2 | * |
| **Monoclonal antibody** | | | | | | | | | | | | | | | |
| Raison 2013 | 2013 | Monoclonal antibody | infliximab | QIDS-SR≥14 | 42.5±18.2 | 10/20 | 30 | 12 | 25 | 15 | 3 | 50%HAMD decrease | HAMD ≤7 at end of treatment | 5 | * |
| Raison 2013 | 2013 | Placebo | Placebo | QIDS-SR≥14 | 44.3±9.4 | 10/20 | 30 | 12 | 28 | 15 | 8 | 50%HAMD decrease | HAMD ≤7 at end of treatment | 1 | * |
| Abbasian 2022 | 2022 | Monoclonal antibody | Adalimumab 5mg/kg + Sertraline 100g/d | HAMD-17≥19 | 35.87±6.07 | 10/8 | 18 | 6 | 15 | 15 | 4 | 50%HAMD decrease | HAMD ≤7 at end of treatment | 3 | * |
| Abbasian 2022 | 2022 | Placebo | Placebo + Sertraline 100mg/d | HAMD-17≥19 | 34.60±9.86 | 8/10 | 18 | 6 | 15 | 10 | 0 | 50%HAMD decrease | HAMD ≤7 at end of treatment | 3 | * |
