# Supplementary material for: Efficacy and acceptability of anti-inflammatory agents in major depressive disorder: a systematic review and meta-analysis
Source: Front Psychiatry. 2024 May 28;15:1407529. doi: 10.3389/fpsyt.2024.1407529 (PMC11165078; doi:10.3389/fpsyt.2024.1407529)
Supplement: Supplementary file 1 [file DataSheet_1.zip › Supplementary Table 5.DOCX]

Table S5. Results of NMA for efficacy and acceptability

| **Efficacy** | **Acceptability** |  |  |  |  |  |  |  |
| --- | --- | --- | --- | --- | --- | --- | --- | --- |
|  |  |  |  |  |  |  |  |  |
| Omega-3 | 0.72 (0.37,1.40) | 1.39 (0.38,5.12) | 1.76 (0.89,3.49) | 0.79 (0.46,1.36) | 2.15 (0.53,8.71) | 1.31 (0.39,4.44) | 1.09 (0.16,7.21) | 1.09 (0.83,1.43) |
| 0.90 (0.29,2.79) | NSAIDs | 1.94 (0.47,7.98) | **2.45 (1.02,5.90)** | 1.10 (0.51,2.39) | 2.99 (0.66,13.48) | 1.82 (0.48,6.97) | 1.52 (0.21,10.88) | 1.52 (0.82,2.81) |
| **0.06 (0.00,0.82)** | 0.07 (0.00,1.04) | Pioglitazone | 1.26 (0.31,5.22) | 0.57 (0.15,2.21) | 1.54 (0.24,10.04) | 0.94 (0.16,5.38) | 0.78 (0.08,7.52) | 0.78 (0.22,2.80) |
| 1.44 (0.48,4.35) | 1.60 (0.41,6.26) | **23.19 (1.58,339.38)** | Minocycline | **0.45 (0.20,0.99)** | 1.22 (0.27,5.53) | 0.74 (0.19,2.86) | 0.62 (0.09,4.46) | 0.62 (0.33,1.16) |
| 0.90 (0.21,3.96) | 1.00 (0.18,5.49) | 14.48 (0.84,250.77) | 0.62 (0.12,3.27) | NACs | 2.72 (0.64,11.61) | 1.66 (0.46,5.96) | 1.38 (0.20,9.49) | 1.38 (0.86,2.21) |
| 0.82 (0.17,3.93) | 0.91 (0.16,5.15) | 13.16 (0.71,242.91) | 0.57 (0.10,3.23) | 0.91 (0.12,6.78) | Monoclonal antibody | 0.61 (0.10,3.76) | 0.51 (0.05,5.17) | 0.51 (0.13,2.01) |
| 0.42 (0.09,1.94) | 0.46 (0.08,2.69) | 6.68 (0.37,119.11) | 0.29 (0.05,1.59) | 0.46 (0.07,3.27) | 0.51 (0.06,3.97) | Statins | 0.83 (0.09,7.65) | 0.83 (0.25,2.74) |
| 0.18 (0.01,2.40) | 0.20 (0.01,3.05) | 2.87 (0.08,101.28) | 0.12 (0.01,1.85) | 0.20 (0.01,3.50) | 0.22 (0.01,4.09) | 0.43 (0.02,7.80) | Corticosteroids | 1.00 (0.15,6.50) |
| 1.77 (0.98,3.21) | 1.97 (0.70,5.53) | **28.50 (2.32,350.58)** | 1.23 (0.48,3.18) | 1.97 (0.51,7.63) | 2.17 (0.49,9.55) | **4.27 (1.04,17.56)** | 9.92 (0.79,124.24) | Placebo |

Data are ORs (95% CI) in the column-defining treatment compared with the row-defining treatment. In the efficacy results (blue box), ORs higher than 1 favor the column-defining treatment, and ORs lower than 1 favor the row-defining treatment. In acceptability results (green box), ORs lower than 1 favor the column-defining treatment, and ORs higher than 1 favor the row-defining treatment. Significant results are in bold and underlined. NSAIDs: nonsteroidal anti-inflammatory drugs; NACs: N-acetylcysteines.
