# Supplementary material for: Efficacy and acceptability of anti-inflammatory agents in major depressive disorder: a systematic review and meta-analysis
Source: Front Psychiatry. 2024 May 28;15:1407529. doi: 10.3389/fpsyt.2024.1407529 (PMC11165078; doi:10.3389/fpsyt.2024.1407529)
Supplement: Supplementary file 1 [file DataSheet_1.zip › Supplementary Table 6.DOCX]

Table. S6: SUCRA value for efficacy and acceptability ranking.

| **Treatment** | **Efficacy** | **Acceptability** |
| --- | --- | --- |
| Placebo | *10.9* | 49.7 |
| Omega-3 | 37.1 | 59.8 |
| NSAIDs | 44.3 | **81.6** |
| Pioglitazone | **93.3** | 41.3 |
| Minocycline | 24.1 | *22.1* |
| NACs | 44.5 | 77.3 |
| Monoclonal antibody | 46.3 | 22.7 |
| Statins | 69.1 | 43.0 |
| Corticosteroids | 80.3 | 52.3 |

The surface under the cumulative ranking curve (SUCRA) value is a representative number of the overall ranking, and a higher SUCRA value indicates a higher probability. The highest values of SUCRA are in bold, and the lowest values of SUCRA are in italic font. NSAIDs: nonsteroidal anti-inflammatory drugs; NACs: N-acetylcysteines.
